# Community acceptability of Seasonal Malaria Chemoprevention of morbidity and mortality in young children: A qualitative study in the Upper West Region of Ghana

**DOI:** 10.1371/journal.pone.0216486

**Published:** 2019-05-17

**Authors:** Samuel Chatio, Nana Akosua Ansah, Denis A. Awuni, Abraham Oduro, Patrick O. Ansah

**Affiliations:** Navrongo Health Research Centre, Navrongo, Ghana; University of Alabama at Birmingham, UNITED STATES

## Abstract

**Background:**

Malaria remains a major public health problem, especially in sub-Sahara African countries. The Malaria Control Program of Ghana has implemented Seasonal Malaria Chemoprevention (SMC) intervention in the Upper West Region in 2015. This preventive drug has been recommended by WHO as very safe and effective in preventing malaria in children under five years. This study assessed community acceptability of the SMC intervention in the Lawra district of Northern Ghana.

**Methodology:**

This was a qualitative study where focus group discussions and in-depth interviews were conducted with community-based health volunteers and parents whose children received the SMC drug. Purposive sampling method was used to select study participants. The interviews were recorded with consent of study participants. All interviews were transcribed and coded into emergent themes using Nvivo 10 software before thematic content analysis.

**Results:**

Study participants perceived that the introduction of the SMC intervention in the area had helped to reduce the prevalence of malaria among children less than five years of age. Parents held the view that the drug was very good in preventing malaria. The results also showed high acceptability of the SMC intervention by parents and other community members. Parents reported that they were willing to allow their children to receive the drug and wished the intervention could continue in the district for children to continue to benefit. Nonetheless, negative attitude on the part of few parents made them not to allow their children to receive the drug.

**Conclusion:**

The interpretation of our data showed high acceptability of the SMC by stakeholders in the study area. However, intensive and continued health education on the benefits of the SMC drug could help to further improve acceptability of the program.

## Introduction

Malaria is the main public health problem in the world especially in sub-Sahara Africa. In 2016, about 216 million clinical malaria cases with 445,000 deaths were reported globally [1]. About 90% of malaria cases with 91% deaths were recorded in Africa alone in 2016 [1]. Some population groups are at higher risk of being infected with malaria than others. The most affected groups are infants, children under five years, pregnant women and HIV patients [1]. Several interventions such as Intermittent Preventive Treatment of Malaria in Infants (IPTi) and Pregnant Women (IPTp), Indoor Residual Spraying (IRS), the use of long lasting Insecticide Treated bed Nets (ITNs) and early diagnosis and treatment of confirmed malaria cases with effective artemisinin-based combination therapies have been introduced to help reduce the prevalence of malaria in Ghana [1, 2]. Despite these interventions, malaria is still endemic and a leading cause of morbidity and mortality especially in children and pregnant women in Ghana [3]. In 2015 alone, the total number of malaria admissions was 409,947 with 1,137 deaths respectively. Of these deaths 1, 037 occurred among children under 5 years of age [4].

In 2015, the Malaria Control Programme of Ghana implemented the Seasonal Malaria Chemoprevention (SMC) intervention in the Upper West Region of Northern Ghana. The region is situated at the North western part of Ghana, within the Sahel sub-region where most childhood mortality and morbidity from malaria occurs during the rainy season [5]. This is a preventive drug that has been recommended by the WHO as an additional tool which has been shown to be effective, safe and feasible in preventing malaria among children under five years of age [6]. In Ghana, the National Malaria Control Program (NMCP) has used Sulfadoxine pyremethamine and amodiaquine combination for the implementation of the program in the Upper West Region with the main purpose of reducing morbidity and mortality in children less than five years of age in the area [7, 8, 9].

The SMC intervention was introduced in the Region where all children under five years were expected to receive Sulfadoxine pyremethamine and amodiaquine combination. The intervention used the services of Community-Based Health Volunteers supervised by district and sub-district health workers. Evidence suggests that community acceptability and involvement in health intervention programs at the community level is crucial for a successful implementation of these programs [10, 11]. Therefore, the objective of this qualitative study was to assess the community acceptability of the SMC intervention in the Lawra district of the Upper West Region of Northern Ghana. This has helped to provide evidence-based information for scaling up of SMC implementation in other regions and districts in Ghana.

## Methods

### Ethical consideration

Ethical approval for the study was received from the Navrongo Health Research Centre Ethics Review Committee. Written informed consent was obtained from all study participants prior to their participation in the study. Data collectors read and translated the consent forms into the local language for the participants. All participants were informed about the purpose and procedure of the study, their right to refuse or withdraw from the study and the confidentiality of the information collected. To ensure confidentiality, personal identification numbers were assigned to participants instead of their names. Consent was also obtained from study participants for the findings to be published in a peer review journal.

### Study site

The study was conducted by the Navrongo Health Research Centre in the Lawra District of the Upper West Region. The District is one of the Eleven Districts in the Upper West Region. It lies in the North Western corner of the Upper West Region between Latitudes 20 25"W and 2°45"W and Longitudes 10°20" N and 11°00"N. The Administrative capital of the district is Lawra. The District shares boundaries with Nandom District to the North, to the south with Jirapa District, to the west with Ivory Coast and to the East with lambussie-karni. The District has a population of about 100,929 according to the 2010 Ghana National Population and Housing census results. This is about 15.2% of the Region’s total population of 576,583 [12]. The district has five health centres, 10 CHPS compounds, three pharmacy shops, one clinic and one district hospital, which provide health care services to the people in the area [13].

### The implementation of the SMC intervention in the study area

Following the recommendations by the WHO for SMC to be administrated to children under-5 years of age, all eligible children were given full treatment doses of sulfadoxine–pyrimethamine (SP) and amodiaquine (AQ) at monthly intervals from July to November 2015 in the Upper West Region. A full dosed child was given four courses of the treatment. The recommended drug (AQ-SP) was administered to each child as directly observed therapy as scheduled in each cycle. Community-Based Health Volunteers were used to administer the drug with supervision from district and sub-district health workers. Health volunteers visited each household and the target children were given the required number of doses of the anti-malarial per the scheduled and the first dose is directly observed. Each caregiver then administers the rest of the 2 days dose. A passive pharmaco-vigilance system was put in place to record all adverse events. However for the children in this study which was done in Lawra, one of the districts of the pilot region, an active pharmaco-vigilance system was established to capture adverse drug reactions in children who received SMC in the Region. The intervention was carried out in four rounds in all 11 districts in the Region. In addition, several announcements were made at the local radio stations within the Region prior to the implementation of the SMC programme. Meetings and durbars were also held with community members to explain the rationale of the programme to them. The people were reacting to the side effects of the drugs.

### Sample size

This was a qualitative study where In-depth Individual Interviews (IDIs) and Focus Group Discussions (FGDs) were conducted with community members. The IDIs were conducted with community-based health volunteers involved in the program and mothers whose children received the SMC drug. The FGDs were conducted with mothers and fathers with children less than five years in selected communities in the study area. Ten IDIs were conducted with the health volunteers while twenty IDIs were conducted with mothers. Eight FGDs were conducted with mothers and fathers with children less than five years of age.

### Sampling of communities and study participants

Simple random sampling technique was used to first select ten communities for the study. This was done by writing the names of all the communities in the area on a piece of paper each and concealed by wrapping the papers. Two people randomly selected the first ten communities, which were used for the interviews. Purposive sampling method was used to select participants in the study communities for the interviews. Study participants comprised two mothers whose children had received the SMC drug and one health volunteer from each of the ten communities.

Mothers and fathers with children less than five years in four out of the ten study communities were purposively selected for the focus group discussions. Two group discussions (one for mothers and one for fathers) were organised in each of the four communities selected. Each group was made up of a minimum of 8 and a maximum of 12 members. The participants shared their views on the usefulness and the effectiveness of the drug in preventing malaria in children under five years. Their views were also sought on the acceptability of the SMC intervention introduced in the area.

### Recruitment and training of data collectors

Four graduates and two postgraduates (the first and third authors) all of whom are health research officers conducted the interviews. Four of them were males while two were females. All data collectors were all trained intensively for one week before the interviews were conducted. The highly interactive training consisted of an overview of the study with emphasis on the main aim of the study, the design of the study, techniques and strategies in qualitative interviewing, with participants encouraged to ask relevant questions throughout. Data collectors were also taken through the interview guides first in English and then translated into the main local language of the study area. At the end of the training, a pre-test was conducted in order to help finalize the interview guides before data collection.

### Data collection technique

Face to face appointments were booked with all the study participants on the date, time and venue before the interviews were conducted. The interviews were conducted at various meeting points at the community level. The interview participants were both male and female community members in the study area. The interviews were audio tape recorded with the consent of participants after they were told about the rationale of the study. The IDIs lasted for about 35 minutes while the FGDs lasted for about 1 hour. All the interviews were conducted in the local language. All the potential study participants who were contacted agreed to participate in the study. Field notes were taken to serve as backup in case the recording was not done well.

### Data processing and analysis

All the interviews were transcribed verbatim after repeatedly listening to the recordings. A codebook was developed based on the objectives of the study to guide the data coding process. The transcripts were then uploaded onto QSR Nvivo 11 software to facilitate data management and coding [14]. Data coding was done simultaneously with data collection to ensure that thematic saturation was monitored. Also, to ensure a fair interpretation of the data, the transcripts were initially coded by two researchers independently. The coding process involved a critical review of each transcript to identify emerging themes from the data. The two coders then met to compare their independently-identified themes during data coding. They revolved divergence by re-reading the relevant sections of the transcripts together, and agreed on the best fit interpretation of the data. The major and sub-themes are discussed below, supported by relevant quotes from the transcripts.

## Results

### Prevalence of malaria among children under five years of age

The prevalence of malaria was perceived to have reduced among children under five years in the study area mainly because of the introduction of the SMC intervention. Most of them were of the view that the introduction of the SMC intervention in the district had helped to reduce the burden of malaria among children less than five years of age. The use of Insecticide Treated Nets (ITNs) and the indoor residual spraying were also reported by some of the participants as other factors responsible for the reduction. Views expressed were similar in both the IDIs and the FGDs conducted with community members. In their own words study participants gave examples on what has contributed to the reduction of malaria prevalence in the area in the follow quotes:

*“It is decreasing because of the drug (refers to SMC drug) they are giving to our children and our rooms they came to spray*. *These are making malaria cases to reduce here” Also*, *the mosquito nets that we are using help to prevent our children from getting malaria”* (IDI-18 year old mother)

*“The way children were getting sick all the time whenever we take them to the hospital they normally check and say it is malaria*. *For about 4 months now children are not getting sick like that and even if a child is sick and you take him/her to hospital*, *it is not malaria maybe the child is lacking water*. *That is why I said malaria has reduced*” (A mother, FGD participant)

*“It is the SMC drug they give them (refers to children)*. *This is because the SMC drug was given to children between the ages of three months to 59 months and those ages are the most vulnerable and so when they are being affected*, *some of them die but with the help of the SMC*, *malaria cases have reduced in the community”* (IDI-29 year old health volunteer)

### Knowledge on the purpose of the SMC intervention

We solicited the views of stakeholders on the purpose for the SMC drug being given to children under five years. Varied opinions were expressed by study participants in the discussions. Most of them were of the view that the drug was given to protect children from getting malaria. Others also perceived that the drug was given to enable children eat very well and be healthy.

*“It is given to the child to protect him from diseases like malaria*, *the malaria that worry the children…*.*”* (IDI-28 year old mother, Berwong)

*“They have realized that this region is having the highest rate of malaria infection that is why they brought it here to protect the children from malaria infection”* (IDI-35 year old health volunteer, Bagri)

*“It is to protect the child from malaria and also to treat a child that is suffering from malaria*. *Since it protects the child against malaria*, *the child doesn`t fall sick and that makes the child strong and healthy”* (IDI-37 year old mother, Kolbugnuor)

A mother was also of the opinion that the drug was given to prevent diarrhea and polio diseases. In her own words, she shared her views this way on the issue in one of the FGDs:

*“hmmm*, *the drug has also protected our children from diarrhea and polio diseases”* (FGD-mothers, Gbier)

### Patients’ adherence and completion of dosages

We wanted to understand the level of adherence on the use of the drug and so we posed a question to find out from parents whether their children took the full treatment course. The majority of the mothers said that their children completed the require number of doses given them. A 31year old mother shared her views this way on the issue in the following conversation:

*Q*: *Did your child take all the medicine given him?*

*R*: *Yes, he has taken it for 4 times*

*Q*: *Was your child able to take the drug four times without jumping (skipping) a day?*

*R*: *Yes he never jumped a day*. *He took all the medicine throughout* (IDI-31 year old mother, Tuma)

However, few of the parents report that their children were not able to complete the require doses. Some of them reported that their children took it for only two times while others took it for three times out of the required four times they were supposed to receive the SMC drug. The explanation given by participants was that their children were not qualified at the time they started given the drug and for that reason such children were not given the complete dosage. Other reasons given by mothers for children not completing the required dosages included forgetfulness and not being at home because of work or other personal reasons. A 26 year old mother expressed her opinion this way in one of the IDIs:

*“Because the last day we were having funeral*, *so I forgot to give it to him*. *The following day I asked the guy (refers to the health volunteer) who was here and he said since he could not take it the previous day that he can’t take it again”* (IDI-26 year old mother, Eremon Tangzu)

The health volunteers, on the other hand, reported that some of the mothers had problems giving the medication to their children when the intervention started. They explained that they (volunteers) had to give the medicine to the children for the first and second rounds. They said that because it was in the farming season, some of the mothers were not at home for their children to be given the medication. They added that some of the children did not like to take the drug because of the bitter taste. The following quotes from the health volunteers illustrate these points:

*“…we the volunteers give the drugs*. *We gave it to the children on the first and second rounds*. *It was on the third round that the mothers themselves complained that we are tired*, *so we gave it to them and still went round to monitor*. *Sometimes the children do not allow their mothers to give the drugs so we have to intervene”* (IDI-45 year old health volunteer, Gbier)

*“Yeah majority would take it but a few could not*. *You know how children are*, *because of the bitter taste of the drug*, *some children don’t usually want to take it*. *But those that do not accept to take it*, *we let the mothers put in TZ and the child takes it*….” (IDI-29 year old health volunteer)

### Experience of side effects

Views were also solicited from parents on the side effects their children had experienced from using the SMC drug and diverse views were expressed by study participants on the issue. While some of them said their children did not experience any side effects, others reported various side effects their children had experienced when they took the drug. The most commonly reported side effects were diarrhea, body weakness and vomiting. They said that the children had these side effects mostly on the first and second days when they took the drug. The views of study participants are exemplified in the following quotes from the interviews:

*“When my child takes this drug he is always healthy*, *he never took it and got weak*, *his body became stronger than when he did not take the drug”* **(**FGD-mother, Tanziir)

*“It is the diarrhea and the vomiting that I can say*: w*hen my child took the medicine for the first two days*, *he vomited…*.*”* (FGD -father with child under five, Zambo)

*“The first time he took the drug*, *he was weak and when I took him to the clinic*, *they told me not to be afraid because the drug is strong (refers to the components of the drug being high) that is why he is weak*. *They encourage me to still continue to give him and that it will go*. *When I also continue giving him the drug*, *it [the side effects] stopped and he became active and was okay”* (IDI 31 year old mother, Eremon Tangzu)

*“There is vomiting*, *itching or the child becomes weak after taking the medicine*. *If a child was having the disease already he/she will run diarrhea*, *or experience body itching…*.*”* (IDI-35 year old volunteer, Tuma)

### Community acceptability of the SMC intervention

Views of study participants were solicited regarding the level of acceptability of the SMC intervention. The results showed that acceptability was very high among parents and other community members in the study area. Most of the participants said they were happy that their children took the drug. They maintained that nobody forced them to give the drug to their children and that they did not regret giving it to the children. They reported that they were happy about the intervention and they were willing to continue to allow their children to receive the SMC drug. They said it was their wish that the implementers of the program would allow it to continue in their communities for many other children especially those yet to be born to also receive the drug. Similar views were expressed by mothers, fathers and health volunteers on acceptability of the SMC intervention. Several excerpts show how stakeholders in the study area describe their happiness and willingness to accept the SMC intervention:

*“We are happy that they have brought this drug to help our children from getting malaria*. *We have newborns and if they stop what are they going to do*? *They should continue to bring it (the drug) for us”* (FGD, fathers, Tanziir)

*“The drug they gave to my child is very good*. *He has never been to the hospital again ever since he took it (refers to the SMC drug)*. *If they bring it another time to help us I will be very happy to collect it for my child”* (IDI-30 year old mother, Eremon Tangzu)

*“What I have to say about this drug is that the drug is good; the drug is powerful (effective) it has the ability to chase malaria away (meaning to prevent)*. *They should continue to give it to the children”* (IDI-35 year old health volunteer, Bagri)

*“Yes*, *I will still give it to my child because it is very helpful in terms of my child`s health*. *It protected him against malaria and we are all happy about it*. *We thanked those who knew that there were children here who needed help and have taken it upon themselves to bring this drug to help our children”* (IDI 20 year old mother, Berwong)

### Perceptions of other family members and friends

Views were also solicited from study participants about their husbands and other family members’ perceptions and attitudes towards the SMC intervention. All the mothers said that the attitude of their husbands and other family members was positive. They explained that their husbands had no problem concerning the use of the drug because they knew the drug was given to improve the health status of the children. They added that their husbands encouraged and sometimes reminded them to give the drug to the children. Similar views were held by the fathers who were part of the interviews.

*Q*. *What does your husband say about the SMC drug given to prevent children from getting malaria?*

*R*. *He supports the intervention*, *whenever they brings it (the drug)*, *he is always eager for me to give it to the child* (IDI 27 year old mother, Newtown)

*R*: *Ooo*, *for us there is no problem because we have seen that they brought the drug to help our children to be free from malaria…*. *So the men in this community have no problem with their wives for given the drug to the children”* (FGD, father, Tanziir).

*R*: *As for my husband*, *he usually asks me whether I have given the child the drug*. *If I say no*, *then he gets angry at me for me to give the child the drug*. *So he knows that the drug is very helpful to our child’s health”* (IDI 30 year old mother, Bagri)

The health volunteers confirmed the willingness of parents to give the drug to their children. They said that parents believed that the drug was very good and had reduced the burden of malaria in the area. They added that parents who initially doubted the efficacy of the drug also accepted and agreed to give it to their children because of the testimonies shared by their colleagues on how good the drug was in preventing malaria.

*“Yes they are willing to give the drug to the children because there are a lot of testimonies that their friends or colleagues testified on the effectiveness of the drug…*.*”* (IDI-45 year old health volunteer)

*“Yes*, *even this month some of them were asking whether the drug will be brought or not*. *They asked whether the exercise will continue again”* (IDI 35 year old health volunteer)

However, some of the volunteers reported that few of the mothers were not comfortable with the side effects presented by their children, which nearly discouraged them. The health volunteers said that they had some difficulties with some of the mothers at the initial stages of the implementation processes but when they continued with the health education on the importance and the need for them to allow their children to receive the drug, they accepted it.

*“In some places*, *some people tried rejecting the drug because of the side effects*. *One of the mothers said the drug was too hard and that made the child weak*, *but upon explaining further*, *she accepted and gave it to the child”* (IDI- 35 year old health volunteer, Newtown)

*Yes*. *It was the beginning that some people tried to be difficult but when we educated them on the importance of the drug*, *they accepted it*. (IDI- health volunteer, Kolbugnuor)

Despite the few challenges mentioned by the health volunteers, all the mothers who took part in the interviews said that they would recommend the drug to their relatives and friends to give to their children. They also recommended for the drug to be made available to children in other Regions and districts.

*“I will tell my colleagues how this drug is helpful to the children and has taken away petty diseases from the children*. *It has also helped reduced our movement to the hospital frequently for treatment of malaria and so she should try and give it to the child”* (IDI-26 year old mother, Eremon Tangzu)

*“They should continue to other districts for malaria cases to reduce*. *This is because monies that are always used by the government to purchase anti malaria drugs in our hospitals will be channeled to other areas”* (FGD, father, Bagri)

*“I will advise all mothers that they should try and collect these drugs for their children in order to reduce malaria in the country”* (IDI 31 year old mother, Kolbugnuor)

Interestingly, a mother had this to say on whether the drug should be sent to other districts or not. As she put it:

*“Yes*, *if only it is enough for them to give us and still give to other districts*, *there is no problem otherwise*, *they should continue to give to our children here because the drug has a lot of benefits for our children”* (IDI 29 year old mother, Newtown)

## Discussion

Adherence to treatment regimens is the extent to which patients use medication as prescribed to them by health care providers [15]. It is very important that people who are given medications including malaria preventive medications use them according to what they have been told by health providers [16]. A recent study in Kenya reported a fifty-three (53%) non-adherence to the use of malaria treatment [17]. Various factors such as side effects, bitter taste of drugs, forgetfulness and lack of health information on proper use of medications influence patients’ adherence to medicines [18, 19]. Perceived cure of illness after initial dose also affects adherence to the use of medicines [18, 20, 21]. In this study, adherence was generally high. The health education given to parents and the benefits of the drug to children were the reasons for the high level of adherence of the SMC drug in the study area.

Though most of the children completed their medications, a few of the parents in our study reported that their children could not complete the require dosages. There are various factors that influence patients’ adherence to the use of medications, with side effects experienced by patients with malaria who use ACTs contributing greatly to non-adherence [18]. The inability of people already with malaria to contain side effects such as diarrhea, body weakness, vomiting and itching made them to stop using malaria medicines half way through the treatment regimen [18, 19, 20]. Likewise, in this study, a few of the parents who were not comfortable with such side effects presented by their children stopped giving the drug to them. This is also consistent with earlier study where side effects were reported as some of the reasons why community members did not take part in mass drug administration for lymphatic filariasis in sub-Saharan Africa [22, 23, 24]. In addition, little or no knowledge of possible side effects contributed greatly to non-adherence to the use of medicines including malaria medicines [16]. It is therefore possible in this study that the few parents whose children could not complete the SMC drug because of side effects did not have enough health education on the likely side effects of the drug. Some parents entertained fears when their children experienced these side effects, which perhaps discouraged them from allowing their children to complete the require dosages. It is important to incorporate messages on how to manage these side effects into the whole health education package for community members to understand that these side effects are for a short term and not clinically harmful as demonstrated in current literature [22, 23]. Forgetfulness on the part some mothers to give the medication to their children was also reported as one of the factors contributing to non-adherence. Most of the people in the study area are farmers and a small number of parents indicated they forgot to administer the drug because of the demands of farming. In a study to assess compliance to Mass Drug Administration (MDA) Programme for Lymphatic Filariasis Elimination in Ghana, people who were either absent from their homes on the day of the MDA or at the time of the drug distributors’ visit was reported as one of the factors affecting drug intake during mass drug administration progrmames [24].

Our study results showed how overall collateral benefit accrues as a result of the implementation of the SMC intervention in the study area. Stakeholders perceived that the prevalence of malaria in the study area had gone down compared to previous years. The SMC drug showed 83% reduction in clinical attacks of malaria and 77% reduction in severe malaria outcomes [25, 26]. In this study, participants believed that the drug had contributed in reducing the prevalence and burden of malaria among children less than five years in the area, and were generally very willing to allow their children to receive the drug. It is not surprising therefore that parents and other community members expressed their gratitude to the implementers of the program and wished the program would continue. It is clear that parents in the study area placed a high premium on the health of their children and would therefore support any program in the community aimed at improving the health of these children. Acceptability of the SMC intervention was reported to be very high among parents and other stakeholders who were interviewed in this study.

Though, majority of the participants accepted the intervention, views expressed by some of the participants suggested that very few of the parents failed to give the drug to their children. It is human nature that not everybody would appreciate and behave well towards public health programs or interventions. It was not therefore surprising that few parents could not allow their children to use the drug. Misconceptions about free things not being good also affected the acceptability of the SMC drug by few of these parents. In other studies, it is reported that community members were concern about the safety and efficacy of malaria medications [21]. It is possible that because the drug was given free to the children, a small number of parents doubted its effectiveness.

## Conclusion

The study has shown the overall collateral benefit as a result of the implementation of the SMC intervention in the study area. Parents perceived that the SMC drug had contributed in reducing the burden of malaria in the area. Therefore, acceptability on the program was reported to be very high by parents and other stakeholders. It is believed that parents placed a high premium on the health of their children and would therefore support the SMC intervention and any other program in the community aimed at improving the health of their children. However, negative attitude and lack of trust by few parents meant they showed little interest in the intervention. Therefore, intensive education on the SMC intervention with focus on the importance of the drug, the likely side effects and the need for parents to allow their children to receive the drug could further improve the smooth implementation of the program in other areas.

## Supporting information

S1 File Dataset. (ZIP)
