# Supplementary material for: Community acceptability of Seasonal Malaria Chemoprevention of morbidity and mortality in young children: A qualitative study in the Upper West Region of Ghana
Source: PLoS One. 2019 May 17;14(5):e0216486. doi: 10.1371/journal.pone.0216486 (PMC6524792; doi:10.1371/journal.pone.0216486)
Supplement: S1 File — (ZIP) [file pone.0216486.s001.zip › Study data set-Nvivo coding/Acceptability of the SMC interventiuon.docx]

**Mothers views on continuation of the program**

[<Internals\\IDIs health workers\\IDIs mothers\\IDI 18 year old mother-Tanziir>](d9286b71-0d0b-4b69-a3d3-30b1fce77d91) - § 1 reference coded [2.41% Coverage]

Reference 1 - 2.41% Coverage

Q: But do you think this drug they should still continue to give to children or they should stop?

R: They should bring it for them.

Q: Should they send it to other places or not.

R: They can send it all the places, because it is helping.

Q: Like what help?

R: Like the child health.

[<Internals\\IDIs health workers\\IDIs mothers\\IDI 20 yearold mother-Berwong1>](c8dc56f9-1da6-4c14-88d3-30b1fe3e4370) - § 1 reference coded [4.64% Coverage]

Reference 1 - 4.64% Coverage

Q: Should they continue to bring the medicine?

R: Yes, they should continue to bring it.

Q: Would you like them to send this medicine to other districts?

R: Yes, but they should continue to bring it to us.

Q: Should they stop bringing it here and sent it to other places?

R: No, we need it here for our children and so they should continue to bring to us and they can also send it to other area for children to take.

[<Internals\\IDIs health workers\\IDIs mothers\\IDI 26 year old mother-Newtown>](d2cb5fdf-6b7b-4d12-b6d3-30b1fe4a2a75) - § 1 reference coded [3.51% Coverage]

Reference 1 - 3.51% Coverage

Q. Should they continue to bring this drug for the children under five or they should stop?

R. They should continue

Q. Should they introduce it in other district?

R. They should sent it to other districts and still bring some to our district

Q. But what if they want to take what belongs to Lawra districts to other district?

R. No, they should still bring them to our district

[<Internals\\IDIs health workers\\IDIs mothers\\IDI 26 yearold mother-Eremon Tangzu (Autosaved)>](dfa639bf-3760-46df-96d3-30b1fe5ad475) - § 1 reference coded [4.45% Coverage]

Reference 1 - 4.45% Coverage

Q: Would you like they should still continue to this drug to the children?

R: Yes.

Q: But would want the drug be sent to other districts?

R: They should go and give to them so their children can also be healthy like our children.

[<Internals\\IDIs health workers\\IDIs mothers\\IDI 27 year old mother-Newtown>](6ada4bad-92df-4de8-93d3-30b1fe7503c8) - § 1 reference coded [6.82% Coverage]

Reference 1 - 6.82% Coverage

Q. Should the intervention continue?

R. Yes

Q. Should it be introduced in other districts?

R. Yes

Q. What of the case that it should be introduced in other district and stop in this district?

R. No, if they should give to us and to other districts as well. I said no because has help us. If only they can give to us and to other districts, then that will be fine by me.

Q. Do you have the belief that your children can get malaria should they stop taking these drugs?

R. Yes

[<Internals\\IDIs health workers\\IDIs mothers\\IDI 28 year old mother-Berwong>](74cb6986-f6d6-4621-99d3-30b1fe80eacc) - § 2 references coded [3.34% Coverage]

Reference 1 - 1.29% Coverage

Q: So do you think the drug they should continue to bring it to the child who are up to the five years?

R: They should continue to give them.

Q: Should they also send it another district?

R: If they can send it there and also continue to give us I will be collecting.

[<Internals\\IDIs health workers\\IDIs mothers\\IDI 30 year old mother-BagriE>](f4681a8b-1b78-4cab-add3-30b1fe8f3281) - § 2 references coded [2.23% Coverage]

Reference 1 - 0.67% Coverage

R. On my part, i will say they should still continue the distribution

Reference 2 - 1.56% Coverage

Q. Should these drugs be extended to other communities or not?

R. Because its about life saving, they can extend to other communities that will also help them**.**

[<Internals\\IDIs health workers\\IDIs mothers\\IDI 30 year old mother-Eremon Tangzu>](99dc5250-fb67-4d4d-8ad3-30b1fea49eb2) - § 1 reference coded [3.97% Coverage]

Reference 1 - 3.97% Coverage

Q: Do you think the intervention should continue?

R: Yes, I want them to come and continue giving us.

Q: But would you like that they should extend it to other districts?

R: Yes, if they send it to other places to help them I will be happy.

Q: But if they take it to that place and stop here?

R: They should continue with us and send some there.

Q: But if it is not enough and suppose to go to one place, would you like it to come to you or the other place.

R: They should bring it to here.

Q: Why do you think they should continue here?

R: Because I’m here and realize the benefits of the drug that why I don’t want it to be taken anywhere.

[<Internals\\IDIs health workers\\IDIs mothers\\IDI 30 year old mother-Gbier>](90c36595-6809-4c06-96d3-30b1fee4e4c2) - § 1 reference coded [2.76% Coverage]

Reference 1 - 2.76% Coverage

Q: Do you think they should continue giving these drugs to the children?

R: Yes

Q: Should they extend the distribution of this malaria drugs to other communities or districts?

R: Yes. They should extend it to all mothers with children under five in Ghana.

[<Internals\\IDIs health workers\\IDIs mothers\\IDI 30 year old mother-Kolbugnuor>](452bf184-0cec-4af6-95d3-30b1feee696f) - § 1 reference coded [7.98% Coverage]

Reference 1 - 7.98% Coverage

Q. Should the drugs continue?

R. Yes

Q. Why should it continue?

R. It should continue because it is protecting our children from malaria

Q. Should it be introduced in other districts and abandon this district?

R. No, they should introduce it in other district and here also

Q. But in the case they want to pick only one district, will you prefer they take it to another district?

R. No, if they take it to other districts it means my child will not get

[<Internals\\IDIs health workers\\IDIs mothers\\IDI 30 year old mother-Newtown>](1950e505-2542-4323-b9d3-30b1fef7ef36) - § 1 reference coded [7.13% Coverage]

Reference 1 - 7.13% Coverage

Q: Will you like them to continue bringing this medicine for your children?

R: Yes.

Q: Will you like them to send this medicine to other districts?

R: Yes, if only it is enough for them to give us and still give to other districts.

Q: Should they stop bringing the medicine now do you think your child will still be protected against malaria?

R: Yes, I believe that the medicine they gave us will continue to protect our children against malaria.

[<Internals\\IDIs health workers\\IDIs mothers\\IDI 30 year old mother-Tuma>](713306ab-8ca2-47cb-96d3-30b1feff1321) - § 1 reference coded [4.81% Coverage]

Reference 1 - 4.81% Coverage

Q: But do you think this drug they should still continue to give to children?

R: In my mind they should still continue, it helps the children to be healthy.

Q: In your mind would you like them to extend it to other places?

R: Yes, we won’t like this community alone to grow if they can take it to other places we like it.

Q: Do you have something that you want to add to what we have already discussed?

R: I have nothing to say but if can continue to bring this medicine to our children I like it.

[<Internals\\IDIs health workers\\IDIs mothers\\IDI 31 year old mother-Eremon Tangzu>](07b07256-641a-4fb8-9fd3-30b1ff147f2e) - § 1 reference coded [3.60% Coverage]

Reference 1 - 3.60% Coverage

Q: Should they continue to bring this drug to the children or they should stop?

R: I will say the drug helped us but if they can continue to give it we could cure every disease from the head to the toe.

Q: Do you want them to take it to some other districts or not?

R: That is it; I don’t know whether our children are completely protected or not. Like we know that they are completely saved from malaria, we can say they should take it to a different district to also help them too. But if our children are not save, “fu ko tuor ko wie yang kyeli kye buoro sanya” (you cannot farm half way and expect returns or pay).

Q: But I still want to if the drug can cover this community and there is excess will you like it to be send to a different district?

R: I will want it that way.

[<Internals\\IDIs health workers\\IDIs mothers\\IDI 31 year old mother-Kolbugnuor>](e249c295-786b-44e2-96d3-30b1ff33714a) - § 2 references coded [2.64% Coverage]

Reference 1 - 0.79% Coverage

R; The medicine is helping us they should continue to bring it.

Reference 2 - 1.85% Coverage

Q; Should they start giving this medicine to other communities?

R; If the medicine is there they should give it to them and still continue to give us.

[<Internals\\IDIs health workers\\IDIs mothers\\IDI 31 year old mother-Tuma>](70adc82b-bbde-47e1-8fd3-31c514093d39) - § 1 reference coded [3.50% Coverage]

Reference 1 - 3.50% Coverage

Q: But do you think this medicine they should still continue to give to children?

R: For me if I get it every day I like it.

Q: In your mind would you like them to extend it to other places?

R: Yes

Q: Do you have something that you want to say in addition to what we have already discussed?

R: What I will say is this for this dry season I don’t know but if they can continue to help us in the raining they should come and help as.

[<Internals\\IDIs health workers\\IDIs mothers\\IDI 32 year old mother-Gbier>](169607a2-22bc-48ff-b4d3-31cc71f08747) - § 1 reference coded [0.86% Coverage]

Reference 1 - 0.86% Coverage

Q: Should they extend it to other districts?

R: yes, but they should not stop with us.

[<Internals\\IDIs health workers\\IDIs mothers\\IDI 34 year old mother-Tuma>](49e33dc3-05d2-443e-b6d3-30b1ff71558e) - § 2 references coded [4.03% Coverage]

Reference 1 - 0.99% Coverage

Q: Do you think they should let this medicine to continue?

R: In my mind I like it that way they should continue to bring it to our children.

Reference 2 - 3.04% Coverage

Q: But in mind do think they should extend it to other places?

R: I agree with that.

Q: What inform your decision to say that?

R: It is the help that it has for me, my children and my family. So I want it extended to other places and they also talk like me.

Q: You said it has helped you and your family, what help is it?

R: It helps in the sense that I collected for child and my sister also collected so it helped all of us.

[<Internals\\IDIs health workers\\IDIs mothers\\IDI 35 year old mother-Bagri>](4d2676eb-d04a-4099-a3d3-30b1ff7f9d5c) - § 3 references coded [5.42% Coverage]

Reference 1 - 0.88% Coverage

Q: To you do you think they should continue to bring the medicine?

R: Yes, to me they should continue to bring it.

Reference 2 - 1.94% Coverage

Q: In your mind do you think they should take it to some other places.

R: Yes, to me they should extent it to other villages the medicine is helping so that diseases can reduce, not we alone, since the diseases can still spread from there and come to us.

Reference 3 - 2.60% Coverage

R: I don’t have anything to say, what I will say is that the medicine should be extended to all the villages and inform all baby mothers not to be afraid it, they should welcome it and collect for their children. This medicine it makes the child strong, it makes the child healthy, you don’t go to hospital frequently like how it used to be.

[<Internals\\IDIs health workers\\IDIs mothers\\IDI 35 year old mother-Tanziir>](f4a9dcf4-f49d-44b0-9bd3-30b1ff9e8f9d) - § 5 references coded [4.14% Coverage]

Reference 1 - 0.34% Coverage

R: They should continue to give them.

Reference 2 - 0.62% Coverage

R: It can safe our children that is why I said they should continue.

Reference 3 - 1.13% Coverage

Q: Should they also extend this drug to other places and give the children?

R: Yes, they should send it to other places to.

Reference 4 - 0.79% Coverage

Q: Why do you say they should send it to other places?

R: It can protect the children.

[<Internals\\IDIs health workers\\IDIs mothers\\IDI 36 year old mother-Bagri>](8687b6b6-f1c5-444b-acd3-30b1ffbfe296) - § 3 references coded [4.00% Coverage]

Reference 1 - 0.48% Coverage

R: Yes, that is my mind, next year it should be given to as again.

Reference 2 - 1.63% Coverage

Q: In your mind do you think they should go beyond Lawra to a place like Nandom and give this medicine to children?

R: Yes, since we are all Ghanaian, if they help every community that will bring good health to all of us.

[<Internals\\IDIs health workers\\IDIs mothers\\IDI 36 year old mother-Ngman-gbil>](d09e840c-5e47-4690-a9d3-30b1ffcbc9dc) - § 1 reference coded [1.10% Coverage]

Reference 1 - 1.10% Coverage

R: They can be giving us and take some to other places and give to those children there so that they will all be healthy.

[<Internals\\IDIs health workers\\IDIs mothers\\IDI 50 year old mother-Ngman-gbil>](2c4c0db0-dba2-4de3-bcd3-30b1ffdc73e0) - § 1 reference coded [1.01% Coverage]

Reference 1 - 1.01% Coverage

R: They should spread it to all other places where there are children so they can give to the children, it helps.

**Mothers views on whether or not they would want their children to use the drug again**

[<Internals\\IDIs health workers\\IDIs mothers\\IDI 20 year old mother-Gbier>](file:///C:\Users\chatio\Desktop\Save%20in%20drive\studies\PK\SMC%20report\Final%20SMC%20report\Mothers\Acceptability\6d8a376a-063d-4b88-94d3-30b1fe15cc3a) - § 1 reference coded [2.37% Coverage]

Reference 1 - 2.37% Coverage

Q: Would you like to give the drug again to your child some years to come to protect him from malaria?

R: Yes, i will.

Q: Can you tell me why you will allow them to give this drug to your child in future?

R: It is because my child does no longer suffer from malaria and minor illness.

[<Internals\\IDIs health workers\\IDIs mothers\\IDI 26 year old mother-Newtown>](file:///C:\Users\chatio\Desktop\Save%20in%20drive\studies\PK\SMC%20report\Final%20SMC%20report\Mothers\Acceptability\d2cb5fdf-6b7b-4d12-b6d3-30b1fe4a2a75) - § 1 reference coded [2.64% Coverage]

Reference 1 - 2.64% Coverage

Q. Would you like to give this drug to your child to protect him from malaria in subsequent years?

R. Yes, I will be willing

Q. Why will you be willing to give it to your child?

R. It is because I do not want my child to be affected by malaria. I want it to protect him from it for me.

[<Internals\\IDIs health workers\\IDIs mothers\\IDI 26 yearold mother-Eremon Tangzu (Autosaved)>](file:///C:\Users\chatio\Desktop\Save%20in%20drive\studies\PK\SMC%20report\Final%20SMC%20report\Mothers\Acceptability\dfa639bf-3760-46df-96d3-30b1fe5ad475) - § 1 reference coded [2.01% Coverage]

Reference 1 - 2.01% Coverage

Q: Would you like to give this same drug to your child some year to come to protect him from malaria?

R: Yes, I will thank them.

Q: Why do you say if they bring it again you will thank them?

R: Because the drug helped us, because our children are not getting sick anymore. My child especially does not getting sick anymore. If I even hear that the drug is at any place I would go for it.

[<Internals\\IDIs health workers\\IDIs mothers\\IDI 27 year old mother-Newtown>](file:///C:\Users\chatio\Desktop\Save%20in%20drive\studies\PK\SMC%20report\Final%20SMC%20report\Mothers\Acceptability\6ada4bad-92df-4de8-93d3-30b1fe7503c8) - § 2 references coded [2.81% Coverage]

Reference 1 - 1.70% Coverage

Q. Will you like in future to accept to give this drug to your children in other to protect them from malaria?

R. They are most welcome. I will accept the drug.

Reference 2 - 1.11% Coverage

Q. Why will you accept the drugs?

R. The drugs have been so helpful in preventing malaria in my children.

[<Internals\\IDIs health workers\\IDIs mothers\\IDI 28 year old mother-Berwong>](file:///C:\Users\chatio\Desktop\Save%20in%20drive\studies\PK\SMC%20report\Final%20SMC%20report\Mothers\Acceptability\74cb6986-f6d6-4621-99d3-30b1fe80eacc) - § 1 reference coded [4.83% Coverage]

Reference 1 - 4.83% Coverage

Q: Would you like if they bring this drug another year that you should collect for your children will collect for them.

R: Yes I will collect for my child.

Q: Why would you collect for him?

R: Because protect my child and protect myself if my child is sick then I’m not having power again the things that I would have been doing can do again but if my child is not sick whatever I want to do can do to make money but if my child is sick and I go to be at the hospital all my work will go cannot be done, but he is now strong running round doing his own thing and I’m also running round, is it not helped me it helped me.

[<Internals\\IDIs health workers\\IDIs mothers\\IDI 30 year old mother-BagriE>](file:///C:\Users\chatio\Desktop\Save%20in%20drive\studies\PK\SMC%20report\Final%20SMC%20report\Mothers\Acceptability\f4681a8b-1b78-4cab-add3-30b1fe8f3281) - § 2 references coded [2.45% Coverage]

Reference 1 - 0.95% Coverage

R. If they bring it again and say that my child is qualified to partake, why not. I will be glad

Reference 2 - 1.50% Coverage

Q. Why will you give the drug to your child provided he qualified?

R. I have seen how this drugs has helped my child, so i believe it will help him again

[<Internals\\IDIs health workers\\IDIs mothers\\IDI 30 year old mother-Eremon Tangzu>](file:///C:\Users\chatio\Desktop\Save%20in%20drive\studies\PK\SMC%20report\Final%20SMC%20report\Mothers\Acceptability\99dc5250-fb67-4d4d-8ad3-30b1fea49eb2) - § 1 reference coded [2.68% Coverage]

Reference 1 - 2.68% Coverage

Q: If they bring this drug again would you like to collect it for your child?

R: Yes.

Q: Why would you like to collect it for him?

R: The benefits I realized, I’m not going to hospital with him regularly as I used to and they said it is malaria. But when he took this drug I don’t go there regularly. That is why I will collect the drug again. Also, malaria disease is not good and I don’t want my child to go to hospital with it again.

[<Internals\\IDIs health workers\\IDIs mothers\\IDI 30 year old mother-Gbier>](file:///C:\Users\chatio\Desktop\Save%20in%20drive\studies\PK\SMC%20report\Final%20SMC%20report\Mothers\Acceptability\90c36595-6809-4c06-96d3-30b1fee4e4c2) - § 1 reference coded [2.38% Coverage]

Reference 1 - 2.38% Coverage

Q: Would you like to give this drug to your children some years to come to protect them from getting malaria?

R: Yes

Q: Why?

R: I will take them for my children due to the benefit that they derived from the previous one.

[<Internals\\IDIs health workers\\IDIs mothers\\IDI 30 year old mother-Kolbugnuor>](file:///C:\Users\chatio\Desktop\Save%20in%20drive\studies\PK\SMC%20report\Final%20SMC%20report\Mothers\Acceptability\452bf184-0cec-4af6-95d3-30b1feee696f) - § 2 references coded [3.87% Coverage]

Reference 1 - 2.37% Coverage

Q. Will you take the drugs for child in subsequent years?

R. Yes, if only my child is within the age range

Q. Why will you accept the drugs for your child?

R. It has helped. My child has been protected from malaria

[<Internals\\IDIs health workers\\IDIs mothers\\IDI 30 year old mother-Newtown>](file:///C:\Users\chatio\Desktop\Save%20in%20drive\studies\PK\SMC%20report\Final%20SMC%20report\Mothers\Acceptability\1950e505-2542-4323-b9d3-30b1fef7ef36) - § 1 reference coded [2.72% Coverage]

Reference 1 - 2.72% Coverage

Q: Will you like to take this medicine for your child next year?

R: Yes.

Q: Why will you take the medicine for your child?

R: For it to protect the child against malaria.

[<Internals\\IDIs health workers\\IDIs mothers\\IDI 31 year old mother-Eremon Tangzu>](file:///C:\Users\chatio\Desktop\Save%20in%20drive\studies\PK\SMC%20report\Final%20SMC%20report\Mothers\Acceptability\07b07256-641a-4fb8-9fd3-30b1ff147f2e) - § 2 references coded [3.52% Coverage]

Reference 1 - 2.71% Coverage

Q: So, would you want to give it to him in the future should in case they bring it again to protect him from getting malaria disease?

R: Yes, I will be happy if they help us again with this drug, it will also help our children.

Q: Why would you want to give it to you child again?

R: For the health reason that I want them to come and help us because the drug is good, it is good for the children and also the parents.

Q: Why do you say it is good for parents?

R: Because if your child is healthy you are also healthy but if your child is not then you as parent will not also healthy.

[<Internals\\IDIs health workers\\IDIs mothers\\IDI 31 year old mother-Tuma>](file:///C:\Users\chatio\Desktop\Save%20in%20drive\studies\PK\SMC%20report\Final%20SMC%20report\Mothers\Acceptability\70adc82b-bbde-47e1-8fd3-31c514093d39) - § 1 reference coded [2.58% Coverage]

Reference 1 - 2.58% Coverage

Q: So, a year to come if they bring that drug for you will you give it to your child?

R: Yes, even if I get them to continue to give us for the whole year I will be happy.

Q: For what reason will give to your child?

R: The child, the way he was and I gave him this medicine good health that he has made me to say that.

[<Internals\\IDIs health workers\\IDIs mothers\\IDI 32 year old mother-Gbier>](file:///C:\Users\chatio\Desktop\Save%20in%20drive\studies\PK\SMC%20report\Final%20SMC%20report\Mothers\Acceptability\169607a2-22bc-48ff-b4d3-31cc71f08747) - § 1 reference coded [1.92% Coverage]

Reference 1 - 1.92% Coverage

Q: would you like to give these drugs to your child a year to come to protect him against malaria?

R: yes

Q: why?

R: The drug has cut down my expenses and my up and down movement to the hospital.

[<Internals\\IDIs health workers\\IDIs mothers\\IDI 35 year old mother-Bagri>](file:///C:\Users\chatio\Desktop\Save%20in%20drive\studies\PK\SMC%20report\Final%20SMC%20report\Mothers\Acceptability\4d2676eb-d04a-4099-a3d3-30b1ff7f9d5c) - § 1 reference coded [1.22% Coverage]

Reference 1 - 1.22% Coverage

Q: Do you think next year you will give your child same medicine if they bring it?

R: Even tomorrow if they bring it I will collect it and give to him again.

[<Internals\\IDIs health workers\\IDIs mothers\\IDI 35 year old mother-Tanziir>](file:///C:\Users\chatio\Desktop\Save%20in%20drive\studies\PK\SMC%20report\Final%20SMC%20report\Mothers\Acceptability\f4a9dcf4-f49d-44b0-9bd3-30b1ff9e8f9d) - § 1 reference coded [1.78% Coverage]

Reference 1 - 1.78% Coverage

Q: If they bring this medicine another year would you agree to collect it for your child or not?

R: I will give him.

Q: Why will you give him?

R: I think it will protect my child from malaria.

[<Internals\\IDIs health workers\\IDIs mothers\\IDI 36 year old mother-Bagri>](file:///C:\Users\chatio\Desktop\Save%20in%20drive\studies\PK\SMC%20report\Final%20SMC%20report\Mothers\Acceptability\8687b6b6-f1c5-444b-acd3-30b1ffbfe296) - § 1 reference coded [1.22% Coverage]

Reference 1 - 1.22% Coverage

Q: So, do you think next year or other years to come they should give the drugs to your child again, or what?

R: Even anytime soon, not even up to a year we want it.

[<Internals\\IDIs health workers\\IDIs mothers\\IDI 36 year old mother-Ngman-gbil>](file:///C:\Users\chatio\Desktop\Save%20in%20drive\studies\PK\SMC%20report\Final%20SMC%20report\Mothers\Acceptability\d09e840c-5e47-4690-a9d3-30b1ffcbc9dc) - § 1 reference coded [1.76% Coverage]

Reference 1 - 1.76% Coverage

R: I will agree that he should collect.

Q: Why would you agree that your child should collect?

R: Because of what he collected and it protects him so for that reason I will ask him to collect.

[<Internals\\IDIs health workers\\IDIs mothers\\IDI 50 year old mother-Ngman-gbil>](file:///C:\Users\chatio\Desktop\Save%20in%20drive\studies\PK\SMC%20report\Final%20SMC%20report\Mothers\Acceptability\2c4c0db0-dba2-4de3-bcd3-30b1ffdc73e0) - § 1 reference coded [1.38% Coverage]

Reference 1 - 1.38% Coverage

R: They will collect, if they bring I let them collect.

Q: Why would you let them collect?

R: I will let him collect to protect him from getting malaria.

[<Internals\\IDIs health workers\\IDIs mothers\\IDI mother-Tanziir>](file:///C:\Users\chatio\Desktop\Save%20in%20drive\studies\PK\SMC%20report\Final%20SMC%20report\Mothers\Acceptability\9368addf-d833-4882-86d3-30b1ffe5f82d) - § 2 references coded [1.13% Coverage]

Reference 1 - 0.31% Coverage

R: Yes, I will collect and give him.

Reference 2 - 0.82% Coverage

R: Because of how it protects my child when I go to hospital they never say it is malaria again.

[<Internals\\IDIs health workers\\IDIs mothers\\IDI-23 year old mother-Berwong>](file:///C:\Users\chatio\Desktop\Save%20in%20drive\studies\PK\SMC%20report\Final%20SMC%20report\Mothers\Acceptability\1361ca8d-5b5d-4820-88d3-3eaa2e45ad3d) - § 2 references coded [1.78% Coverage]

Reference 1 - 0.99% Coverage

R: yes

Q: why will you accept it for protecting her from malaria?

R: because of the difference I have seen in it I know that if I continue to give her this drug it will help her concerning malaria disease.

**Mother opinion on whether or not they would want to recommend the drug to friends**

[<Internals\\IDIs health workers\\IDIs mothers\\IDI 18 year old mother-Tanziir>](file:///C:\Users\chatio\Desktop\Save%20in%20drive\studies\PK\SMC%20report\Final%20SMC%20report\Mothers\Acceptability\d9286b71-0d0b-4b69-a3d3-30b1fce77d91) - § 1 reference coded [2.12% Coverage]

Reference 1 - 2.12% Coverage

Q: Would you tell your friend or someone you are staying with that she shouldn’t collect the drug or to collect for her child?

R: I will tell her to take it for the child. The drug will help her child because he/she would not be sick as it used to be.

[<Internals\\IDIs health workers\\IDIs mothers\\IDI 20 year old mother-Gbier>](file:///C:\Users\chatio\Desktop\Save%20in%20drive\studies\PK\SMC%20report\Final%20SMC%20report\Mothers\Acceptability\6d8a376a-063d-4b88-94d3-30b1fe15cc3a) - § 1 reference coded [1.02% Coverage]

Reference 1 - 1.02% Coverage

Q: Will you recommend this drug to your friend/relative to give it to her child to protect the child from malaria?

R: Yes

[<Internals\\IDIs health workers\\IDIs mothers\\IDI 20 yearold mother-Berwong1>](file:///C:\Users\chatio\Desktop\Save%20in%20drive\studies\PK\SMC%20report\Final%20SMC%20report\Mothers\Acceptability\c8dc56f9-1da6-4c14-88d3-30b1fe3e4370) - § 1 reference coded [1.27% Coverage]

Reference 1 - 1.27% Coverage

Q: Would you encourage a relative or a friend to take this medicine for her child to protect him/her against malaria?

R: Yes.

[<Internals\\IDIs health workers\\IDIs mothers\\IDI 26 year old mother-Newtown>](file:///C:\Users\chatio\Desktop\Save%20in%20drive\studies\PK\SMC%20report\Final%20SMC%20report\Mothers\Acceptability\d2cb5fdf-6b7b-4d12-b6d3-30b1fe4a2a75) - § 2 references coded [4.61% Coverage]

Reference 1 - 2.34% Coverage

Q. Would you recommend these drugs to another mother to give to her child in other to protect him/her from malaria?

R. Yes

Q. Why will you recommend this drug to others?

R. It protects my child from malaria and I want their children to also be protected.

[<Internals\\IDIs health workers\\IDIs mothers\\IDI 26 yearold mother-Eremon Tangzu (Autosaved)>](file:///C:\Users\chatio\Desktop\Save%20in%20drive\studies\PK\SMC%20report\Final%20SMC%20report\Mothers\Acceptability\dfa639bf-3760-46df-96d3-30b1fe5ad475) - § 1 reference coded [1.64% Coverage]

Reference 1 - 1.64% Coverage

Q: Would you recommend this drug to a colleague to give it to the child to protect him/her from getting malaria?

R: I will tell my colleague to collect for the child, because it is good, and it is able to protect children against malaria

[<Internals\\IDIs health workers\\IDIs mothers\\IDI 27 year old mother-Newtown>](file:///C:\Users\chatio\Desktop\Save%20in%20drive\studies\PK\SMC%20report\Final%20SMC%20report\Mothers\Acceptability\6ada4bad-92df-4de8-93d3-30b1fe7503c8) - § 1 reference coded [1.99% Coverage]

Reference 1 - 1.99% Coverage

Q. Will you recommend this drug to a relative or a friend to give to her child to protect him or her from getting malaria?

R. Yes. The drug protects children from malaria so I will recommend

[<Internals\\IDIs health workers\\IDIs mothers\\IDI 30 year old mother-BagriE>](file:///C:\Users\chatio\Desktop\Save%20in%20drive\studies\PK\SMC%20report\Final%20SMC%20report\Mothers\Acceptability\f4681a8b-1b78-4cab-add3-30b1fe8f3281) - § 1 reference coded [2.14% Coverage]

Reference 1 - 2.14% Coverage

R. I will tell my colleagues how this drug is helpful to the children and has taken away petty diseases from the children. It has also helped reduced our movement to the hospital frequently for treatment of malaria in the children, so she should try and give it to the child (IDI30 year old mother Bagri)

[<Internals\\IDIs health workers\\IDIs mothers\\IDI 30 year old mother-Eremon Tangzu>](file:///C:\Users\chatio\Desktop\Save%20in%20drive\studies\PK\SMC%20report\Final%20SMC%20report\Mothers\Acceptability\99dc5250-fb67-4d4d-8ad3-30b1fea49eb2) - § 1 reference coded [2.24% Coverage]

Reference 1 - 2.24% Coverage

Q: But will you tell your colleague mothers if they bring this medicine the fellow should collect and give to his/her child or you say she not collect it?

R: I will say she should accept it for the child.

Q: Why would like your colleague to also collect it for her child?

R: It is helping in fighting the malaria disease in children and they don’t get sick anymore.

[<Internals\\IDIs health workers\\IDIs mothers\\IDI 30 year old mother-Gbier>](file:///C:\Users\chatio\Desktop\Save%20in%20drive\studies\PK\SMC%20report\Final%20SMC%20report\Mothers\Acceptability\90c36595-6809-4c06-96d3-30b1fee4e4c2) - § 2 references coded [3.31% Coverage]

Reference 1 - 1.61% Coverage

Q: Would you recommend or advise your friend/relative to also take these drugs for her child to protect her child from getting malaria?

R: Yes, I will.

[<Internals\\IDIs health workers\\IDIs mothers\\IDI 30 year old mother-Kolbugnuor>](file:///C:\Users\chatio\Desktop\Save%20in%20drive\studies\PK\SMC%20report\Final%20SMC%20report\Mothers\Acceptability\452bf184-0cec-4af6-95d3-30b1feee696f) - § 1 reference coded [3.15% Coverage]

Reference 1 - 3.15% Coverage

Q. Will you recommend these drugs to other mothers to give to their children?

R. Yes. During the period, i even went home and asked my sister inlaw whether she had taken some of the drugs for her child that was at nandom. So i told them to take the drug whenever the opportunity is given them

[<Internals\\IDIs health workers\\IDIs mothers\\IDI 30 year old mother-Tuma>](file:///C:\Users\chatio\Desktop\Save%20in%20drive\studies\PK\SMC%20report\Final%20SMC%20report\Mothers\Acceptability\713306ab-8ca2-47cb-96d3-30b1feff1321) - § 1 reference coded [1.26% Coverage]

Reference 1 - 1.26% Coverage

Q: Can you say someone shouldn’t collect the drug or to collect?

R: I will say the person should collect so all have good health.

[<Internals\\IDIs health workers\\IDIs mothers\\IDI 31 year old mother-Eremon Tangzu>](file:///C:\Users\chatio\Desktop\Save%20in%20drive\studies\PK\SMC%20report\Final%20SMC%20report\Mothers\Acceptability\07b07256-641a-4fb8-9fd3-30b1ff147f2e) - § 1 reference coded [5.00% Coverage]

Reference 1 - 5.00% Coverage

Q: But would you tell a colleague mother to give this drug to her child or not?

R: A colleague mother of mine is just staying by me does give food to the child whenever the volunteer comes around. I told her to always do well and give the child some food that the drug is good, but she told me that they only go round to deceive people. I asked her to look at my child how he now looks compare to the time he was not on the drug.

[<Internals\\IDIs health workers\\IDIs mothers\\IDI 31 year old mother-Kolbugnuor>](file:///C:\Users\chatio\Desktop\Save%20in%20drive\studies\PK\SMC%20report\Final%20SMC%20report\Mothers\Acceptability\e249c295-786b-44e2-96d3-30b1ff33714a) - § 1 reference coded [3.25% Coverage]

Reference 1 - 3.25% Coverage

Q; Will you advice a fellow mother to take this medicine for her child?

R; Yes because it has a lot of benefits.

Q; What are the benefits you will tell her if she takes the medicine for her child?

R; I will tell her it protects the child against fever and malaria.

[<Internals\\IDIs health workers\\IDIs mothers\\IDI 31 year old mother-Tuma>](file:///C:\Users\chatio\Desktop\Save%20in%20drive\studies\PK\SMC%20report\Final%20SMC%20report\Mothers\Acceptability\70adc82b-bbde-47e1-8fd3-31c514093d39) - § 1 reference coded [2.12% Coverage]

Reference 1 - 2.12% Coverage

Q: Can you say someone shouldn’t collect the drug or to collect?

R: No, I can’t say that my colleague should not collect; I will tell her to collect the medicine is helping, my child was like this but now it is this. If I tell her that she may collect the medicine.

[<Internals\\IDIs health workers\\IDIs mothers\\IDI 32 year old mother-Gbier>](file:///C:\Users\chatio\Desktop\Save%20in%20drive\studies\PK\SMC%20report\Final%20SMC%20report\Mothers\Acceptability\169607a2-22bc-48ff-b4d3-31cc71f08747) - § 1 reference coded [2.78% Coverage]

Reference 1 - 2.78% Coverage

Q: would you recommend this drug to your friend to take this drug for her child under five years to protect the child against malaria?

R: yes

Q: why?

R: If my sister child is sick, automatically I will not be able to sleep. I would therefore want them to be protected against malaria.

[<Internals\\IDIs health workers\\IDIs mothers\\IDI 34 year old mother-Tuma>](file:///C:\Users\chatio\Desktop\Save%20in%20drive\studies\PK\SMC%20report\Final%20SMC%20report\Mothers\Acceptability\49e33dc3-05d2-443e-b6d3-30b1ff71558e) - § 1 reference coded [1.93% Coverage]

Reference 1 - 1.93% Coverage

R: Yes

Q: What is reason why you will ask that person to collect the medicine for his/her child?

R: I will tell him/her this medicine they are bringing it has made children ‘’yantulu’’ (illness) to reduce and growth to is going well, their way of thinking to is also good.

[<Internals\\IDIs health workers\\IDIs mothers\\IDI 35 year old mother-Tanziir>](file:///C:\Users\chatio\Desktop\Save%20in%20drive\studies\PK\SMC%20report\Final%20SMC%20report\Mothers\Acceptability\f4a9dcf4-f49d-44b0-9bd3-30b1ff9e8f9d) - § 1 reference coded [2.18% Coverage]

Reference 1 - 2.18% Coverage

Q: Can you tell a colleague when they bring this medicine she shouldn’t collect, or to collect?

R: I will tell her that this medicine is good it has safe my child, he was ill but when they give him this medicine he doesn’t fall ill again.

[<Internals\\IDIs health workers\\IDIs mothers\\IDI 36 year old mother-Bagri>](file:///C:\Users\chatio\Desktop\Save%20in%20drive\studies\PK\SMC%20report\Final%20SMC%20report\Mothers\Acceptability\8687b6b6-f1c5-444b-acd3-30b1ffbfe296) - § 1 reference coded [1.02% Coverage]

Reference 1 - 1.02% Coverage

R: Oh! For that one, many would be informed because of the help that I received from it.

Q: What help?

R: It helps, my child is not sick.

[<Internals\\IDIs health workers\\IDIs mothers\\IDI 36 year old mother-Ngman-gbil>](file:///C:\Users\chatio\Desktop\Save%20in%20drive\studies\PK\SMC%20report\Final%20SMC%20report\Mothers\Acceptability\d09e840c-5e47-4690-a9d3-30b1ffcbc9dc) - § 1 reference coded [1.50% Coverage]

Reference 1 - 1.50% Coverage

R: I will tell them if they bring the drug they should collect it for their children to drink the drug is good, that when I take it for my child it protects him well.

[<Internals\\IDIs health workers\\IDIs mothers\\IDI 50 year old mother-Ngman-gbil>](file:///C:\Users\chatio\Desktop\Save%20in%20drive\studies\PK\SMC%20report\Final%20SMC%20report\Mothers\Acceptability\2c4c0db0-dba2-4de3-bcd3-30b1ffdc73e0) - § 1 reference coded [1.63% Coverage]

Reference 1 - 1.63% Coverage

R: Ok, I always tell the baby mothers who are there that the medicine is good they should collect. Sometimes I can go round and check the children and let them give them the medicine.

**FGDs with father and mothers**

**Continuation of the SMC intervention**

[<Internals\\FGDs\\FGD fathers with children under five-Bagri>](file:///C:\Users\chatio\Desktop\Save%20in%20drive\studies\PK\SMC%20report\Final%20SMC%20report\FGDs\Acceptability\554d2c4c-5aa1-43b0-afd3-2cbc7eb25cc8) - § 1 reference coded [5.36% Coverage]

Reference 1 - 5.36% Coverage

Q. Will you like these drugs to continue and even be extended to other places?

R1. Yes i will be happy. They should continue even to the whole country

R2. In our community, we have seen how helpful it is, so we will need more help and to other communities

R3. They should be extended to the whole country

R4. To me it is good, but they should extend it to we the adults also

R5. I will be happy that it should continue and be extended to other communities

R6. They should satisfy my community first until my community is completed eradicated from malaria before they can think of other communities

R7. They should continue to everywhere

R8. I want it to be extended to the whole country, to the district and to my community. It should be given to both children and adults

R9. They should continue to all communities for malaria cases to reduce. This is because monies that are always used by the government to purchase anti malaria drugs in our hospitals will be channeled to other uses**.**

[<Internals\\FGDs\\FGD fathers with children under five-Tanziir>](file:///C:\Users\chatio\Desktop\Save%20in%20drive\studies\PK\SMC%20report\Final%20SMC%20report\FGDs\Acceptability\12582ff3-0c50-46c7-82d3-3403c9ad39ce) - § 3 references coded [2.85% Coverage]

Reference 1 - 0.74% Coverage

R: We will be happy if they continue to bring it for them (children).

No.4

R: We have newborns if they stop what are they going to do, they should continue to bring it for us.

Reference 2 - 1.80% Coverage

R: The benefit that we have from the drug, for that reason we will say they should continue to bring it.

Q: This drug do you think they should also introduce it in other districts?

R: For the health of the children they also introduce it in other places.

No.8

R: They should continue to bring it here and also introduce it in other places.

No.5

R: If I try to say anything I may be telling lies the truth is what No.8 said.

[<Internals\\FGDs\\FGD mothers with children under five-Bagri>](file:///C:\Users\chatio\Desktop\Save%20in%20drive\studies\PK\SMC%20report\Final%20SMC%20report\FGDs\Acceptability\a0552c32-4da9-42f3-87d3-2cbc7f4d15ae) - § 1 reference coded [2.92% Coverage]

Reference 1 - 2.92% Coverage

Q: Do you think they should continue to give this drug to children under five?

No.4

R: We want them to continue to bring the drug to save us.

No.7

R: It would good to add that of the grownups.

Q: But I think grownups can kill the mosquitoes?

R: But when you slap yourself your body will be paining you.

Q: Would you want this drug should be introduced in other places?

No.3

R: Bring it here first before other places.

Q But if they should leave here and take it there?

R: That is not good we need it here again.

Q: But do you think if they don’t bring it here again your child can get malaria again?

R: That is my mind.

No.2

R: I think that if it is plenty they should give to the whole world, all children in the world.

Q: Across Burkina Faso? Or you wanted to say if they can they should extend it to Accra?

R: If it can cross over to Burkina Faso it will be good, but I meant our government jurisdiction.

[<Internals\\FGDs\\FGD mothers with children under five-Tanziir>](file:///C:\Users\chatio\Desktop\Save%20in%20drive\studies\PK\SMC%20report\Final%20SMC%20report\FGDs\Acceptability\7933f35b-9705-4934-95d3-3403cb0fe604) - § 3 references coded [1.35% Coverage]

Reference 1 - 0.48% Coverage

Q: Should they continue to bring this drug to the children or they should stop.

No.8

R: Continue to bring this drug to our children.

Reference 2 - 0.32% Coverage

No.5

R: What No.8 said, we all agree, they should continue to bring it for our children.

Reference 3 - 0.55% Coverage

No.7

R: They should also introduce it for them to also get good health.

No.2

R: They should also introduce it their children can also get good health.

[<Internals\\FGDs\\FGD mothers with children under five-Zambo>](file:///C:\Users\chatio\Desktop\Save%20in%20drive\studies\PK\SMC%20report\Final%20SMC%20report\FGDs\Acceptability\35ad761a-e06d-40cf-bcd3-3403cb27b45e) - § 1 reference coded [0.57% Coverage]

Reference 1 - 0.57% Coverage

R: They should continue to bring it to the children but they should include the 6 years 7 years own.

Q: Would you like them to introduce it in other districts?

No.2

R: They should continue to bring it to this district.

[<Internals\\FGDs\\FGD-fathers with children under five-Gbier>](file:///C:\Users\chatio\Desktop\Save%20in%20drive\studies\PK\SMC%20report\Final%20SMC%20report\FGDs\Acceptability\72a70c42-22e7-42d9-a8d3-3ea9fb2b287c) - § 6 references coded [2.40% Coverage]

Reference 1 - 0.50% Coverage

Q: Should they continue to bring the drug to the children?

R: They should continue to bring it to them.

Reference 2 - 0.58% Coverage

No.12

R: The mosquitoes are still there so if they can bring it all the times it will be good. That is what I can say.

Reference 3 - 0.28% Coverage

R: They should continue to bring it to help our children.

Reference 4 - 0.45% Coverage

No.9

R: I think if they can cover the whole country it will bring joy to the people of Ghana.

Reference 5 - 0.27% Coverage

No.7

R: If they add that of the adults I will like it.

Reference 6 - 0.32% Coverage

Q: Why will you adults want it?

R: The adult can also get malaria.

[<Internals\\FGDs\\FGD-mothers with children under five-Gbier>](file:///C:\Users\chatio\Desktop\Save%20in%20drive\studies\PK\SMC%20report\Final%20SMC%20report\FGDs\Acceptability\18f67d6c-f146-473c-83d3-3ea9fb47b8e4) - § 2 references coded [1.02% Coverage]

Reference 1 - 0.45% Coverage

R; Yes they should go and give it to other districts.

Reference 2 - 0.58% Coverage

R; They should give it to them and still continue to bring it to us.

**Whether they would want to use it again**

[<Internals\\FGDs\\FGD fathers with children under five-Bagri>](file:///C:\Users\chatio\Desktop\Save%20in%20drive\studies\PK\SMC%20report\Final%20SMC%20report\FGDs\Acceptability\554d2c4c-5aa1-43b0-afd3-2cbc7eb25cc8) - § 1 reference coded [1.40% Coverage]

Reference 1 - 1.40% Coverage

Q. Will you accept these drugs to be given your children in coming years?

R5. Yes we will

R6. Left to me, we will not even want drugs again because we are saved from malaria, the drugs has helped us so much and there is nothing worrying us in this community

[<Internals\\FGDs\\FGD fathers with children under five-Tanziir>](file:///C:\Users\chatio\Desktop\Save%20in%20drive\studies\PK\SMC%20report\Final%20SMC%20report\FGDs\Acceptability\12582ff3-0c50-46c7-82d3-3403c9ad39ce) - § 1 reference coded [0.81% Coverage]

Reference 1 - 0.81% Coverage

R: Even if we get this drug tomorrow we will be very happy with it.

Q: Tomorrow?

No.4

R: We will be happy every year they should be able to bring this drug to save our children from malaria.

[<Internals\\FGDs\\FGD fathers with children under five-Zambo>](file:///C:\Users\chatio\Desktop\Save%20in%20drive\studies\PK\SMC%20report\Final%20SMC%20report\FGDs\Acceptability\903102be-0a51-4b0e-93d3-3403caff3dbb) - § 3 references coded [2.98% Coverage]

Reference 1 - 1.46% Coverage

R; Yes, even if they bring it today we will take it.

Q; Why are you saying you will take it?

R; The medicine is very helpful as far as our children health is concern.

Reference 2 - 1.11% Coverage

R; I will like them to bring the medicine again more especially during the rainy season since mosquitoes breed during that time.

Reference 3 - 0.42% Coverage

R; Yes because the medicine has helped us a lot.

[<Internals\\FGDs\\FGD mothers with children under five-Tanziir>](file:///C:\Users\chatio\Desktop\Save%20in%20drive\studies\PK\SMC%20report\Final%20SMC%20report\FGDs\Acceptability\7933f35b-9705-4934-95d3-3403cb0fe604) - § 5 references coded [2.03% Coverage]

Reference 1 - 0.56% Coverage

No.1

R: they should give to him.

Q: why are you saying they should give to him?

R: when he was sick and they brought the drug he never falls sick again.

Reference 2 - 0.23% Coverage

Q: what sickness was it?

R: any time he was sick it is malaria.

Reference 3 - 0.19% Coverage

No.2

R: I will agree that it should be given to him.

Reference 4 - 0.63% Coverage

Q: why are you saying they should give to him?

R: when he was always getting sick and they brought the drug he never falls sick again so I will they should give to him again.

Reference 5 - 0.42% Coverage

Q: No.3.

R: when they brought I haven’t seen my child sick again if they bring it I will say they should give to him.

[<Internals\\FGDs\\FGD mothers with children under five-Zambo>](file:///C:\Users\chatio\Desktop\Save%20in%20drive\studies\PK\SMC%20report\Final%20SMC%20report\FGDs\Acceptability\35ad761a-e06d-40cf-bcd3-3403cb27b45e) - § 4 references coded [1.14% Coverage]

Reference 1 - 0.47% Coverage

R: Yes.

Q: Why would you like another year if they bring this drug you collect for your child to protect him from malaria?

R: Because it saves our children from a lot of diseases.

Reference 2 - 0.10% Coverage

Q: What diseases?

R: From the malaria.

Reference 3 - 0.22% Coverage

No.7

R: If you bring we will collect.

Q: why would you collect?

R: How it helps me.

Reference 4 - 0.35% Coverage

Q: How did it help you?

R: I never went to Gonbul or Lawra, Gonbul nurses used to know me very well but now they have forgotten of me.

[<Internals\\FGDs\\FGD-mothers with children under five-Gbier>](file:///C:\Users\chatio\Desktop\Save%20in%20drive\studies\PK\SMC%20report\Final%20SMC%20report\FGDs\Acceptability\18f67d6c-f146-473c-83d3-3ea9fb47b8e4) - § 2 references coded [1.66% Coverage]

Reference 1 - 0.94% Coverage

Q; Will you like to take this medicine for your children next year?

NUMBER SEVEN

R; Yes they should bring it.

Reference 2 - 0.72% Coverage

NUMBER TWO

R; They should bring it because it protects our children against malaria.

**Whether they would recommend to friends**

[<Internals\\FGDs\\FGD fathers with children under five-Bagri>](file:///C:\Users\chatio\Desktop\Save%20in%20drive\studies\PK\SMC%20report\Final%20SMC%20report\FGDs\Acceptability\554d2c4c-5aa1-43b0-afd3-2cbc7eb25cc8) - § 1 reference coded [4.88% Coverage]

Reference 1 - 4.88% Coverage

Q. Can you recommend this drug to anybody if the opportunity is offered?

R7. The drug has has helped so much so we can tell others to take when the opportunity is offered them.

R8. I think lawra sub authorities called we the chiefs and volunteers and asked whether the drugs has helped, and we told them how helpful it it to us. We here in the village has accepted the drugs and has also seen the benefits so we still plead if you can even give these drugs to all children not some selected ones

R1. I will tell them to receive the drugs with two hands because it is so helpful

R7. We are still giving birth, even as we speak now, there might be a new born so we will urge you to continue so that those who have not benefited will benefit that will also protect them

R5. I will recommend because if my child is healthy and my neighbors child is not healthy, then that will still bring some disturbances

[<Internals\\FGDs\\FGD fathers with children under five-Tanziir>](file:///C:\Users\chatio\Desktop\Save%20in%20drive\studies\PK\SMC%20report\Final%20SMC%20report\FGDs\Acceptability\12582ff3-0c50-46c7-82d3-3403c9ad39ce) - § 1 reference coded [0.61% Coverage]

Reference 1 - 0.61% Coverage

No.1

R: We will tell our colleagues that the drug is good because we had the benefits of not going to hospital since no child of ours is again.

[<Internals\\FGDs\\FGD mothers with children under five-Bagri>](file:///C:\Users\chatio\Desktop\Save%20in%20drive\studies\PK\SMC%20report\Final%20SMC%20report\FGDs\Acceptability\a0552c32-4da9-42f3-87d3-2cbc7f4d15ae) - § 1 reference coded [1.29% Coverage]

Reference 1 - 1.29% Coverage

Q: Would you tell a colleague to collect for her child or not to?

No.7

R: We will tell our colleague to collect because it will help her and the child concerning malaria disease.

Q: I’m still asking will you tell your colleagues to collect this drug for your children or not to collect for them.

No.7

R: We will tell her to give to avoid the child sickness and the nurses’ harassment at the hospital.

[<Internals\\FGDs\\FGD mothers with children under five-Tanziir>](file:///C:\Users\chatio\Desktop\Save%20in%20drive\studies\PK\SMC%20report\Final%20SMC%20report\FGDs\Acceptability\7933f35b-9705-4934-95d3-3403cb0fe604) - § 5 references coded [3.23% Coverage]

Reference 1 - 0.99% Coverage

R: I will say she should collect the drug for the child to take the drug is helping.

Q: what help does it have?

R: so when the child is sick it is the mosquitoes that normally bite the child to get sick she should give to the child, so that disease will not worry the child.

No.9

R: My child if they bring this drug I will ask them to give him, because my child was always getting sick but when they gave him this drug he is never sick again.

Reference 4 - 0.83% Coverage

R: I will tell her to give the drug is helping.

Q: can you tell me what help it has?

R: my child was always getting sick but when they gave him this drug he is not getting sick again so I will say she should collect for the child.

Reference 5 - 0.65% Coverage

Q: can you make it clear the help that it has.

R: the help is that our children used to get sick regularly but when we started taking this drug we have never experience that again.

[<Internals\\FGDs\\FGD mothers with children under five-Zambo>](file:///C:\Users\chatio\Desktop\Save%20in%20drive\studies\PK\SMC%20report\Final%20SMC%20report\FGDs\Acceptability\35ad761a-e06d-40cf-bcd3-3403cb27b45e) - § 5 references coded [3.70% Coverage]

Reference 1 - 0.76% Coverage

**No.6**

R: There is difference, because the drugs that is in the stores we used them and became fed up no benefit, but this one they brought it has caused all the diseases to go.

Q: What disease?

R: The malaria disease, this one is more powerful than the one we buy and collect from the hospital.

Reference 2 - 0.83% Coverage

Q: So because this one came and you are no more going to hospital that is why you are saying it is more powerful than those drugs?

R: Yes because all those children who used to be sick all the time if you ask their mothers now they normally tell you my child is not getting sick again that is why I said this one is good.

Reference 3 - 1.10% Coverage

R: No you will go and they give you the drug to come and give to the child not up to a week and the child is ill again and when you take the child there that malaria again. And this drug when they brought it they said if you give to the child and child vomits or becomes ill that you should bring the child to the hospital but when I was giving to my child my child never gets sick or vomits so I know this drug is effective.

Reference 4 - 0.58% Coverage

No.2

R: We will tell them if they bring this another time they collect for their children that drug is very good that it will protect their children from getting malaria.

No.3

R: We will say they should collect for them.

Reference 5 - 0.43% Coverage

Q: For what reason would you they should collect for their children?

R: It would protect the children from getting malaria. It also makes the children to grow faster.

[<Internals\\FGDs\\FGD-mothers with children under five-Gbier>](file:///C:\Users\chatio\Desktop\Save%20in%20drive\studies\PK\SMC%20report\Final%20SMC%20report\FGDs\Acceptability\18f67d6c-f146-473c-83d3-3ea9fb47b8e4) - § 1 reference coded [1.08% Coverage]

Reference 1 - 1.08% Coverage

No. 1

R; I will tell my colleagues to take this medicine for their children because it protects the child against malaria.

**IDIs with health volunteers**

[<Internals\\IDIs health volunteers\\IDI 34 year old Health volunteer-Ngman-gbil>](file:///C:\Users\chatio\Desktop\Save%20in%20drive\studies\PK\SMC%20report\Final%20SMC%20report\Volunteers\Acceptability\9792d99e-569d-43a4-aad3-3404088aea56) - § 4 references coded [1.59% Coverage]

Reference 1 - 0.15% Coverage

R: In my mind I will say they should continue.

Reference 2 - 0.49% Coverage

R: In my mind they can do that since I don’t know their strength level, but if they introduce it in other districts I think it would have been good.

Reference 3 - 0.40% Coverage

Q; Do you have something you want to add to all that we discussed?

R: What I have to say is the programme should continue.

Reference 4 - 0.55% Coverage

Q: Why should you say the programme should continue?

R: Because if they don’t continue this programme our children we can’t handle them because of this malaria disease.

[<Internals\\IDIs health volunteers\\IDI 35 year old Health volunteer-Bagri>](file:///C:\Users\chatio\Desktop\Save%20in%20drive\studies\PK\SMC%20report\Final%20SMC%20report\Volunteers\Acceptability\570786a5-e7ba-4707-a3d3-340408a2b74c) - § 1 reference coded [0.31% Coverage]

Reference 1 - 0.31% Coverage

Q: Do you think they should extend this drug to other places?

R: Yes.

[<Internals\\IDIs health volunteers\\IDI 35 year old Health volunteer-Newtown>](file:///C:\Users\chatio\Desktop\Save%20in%20drive\studies\PK\SMC%20report\Final%20SMC%20report\Volunteers\Acceptability\e62ddbb9-5b7b-4af3-aed3-340408b8247e) - § 2 references coded [2.03% Coverage]

Reference 1 - 0.71% Coverage

Q. Should this program continue?

R. God should give grant the suppliers of these drugs drugs to help us

Reference 2 - 1.32% Coverage

Q. Should these drugs be sent to other places ?

R. They should still help us to totally eradicate malaria in our community but still help other places that they think malaria is affecting them

[<Internals\\IDIs health volunteers\\IDI 35 year old Health volunteer-Tuma>](file:///C:\Users\chatio\Desktop\Save%20in%20drive\studies\PK\SMC%20report\Final%20SMC%20report\Volunteers\Acceptability\ec8b1f3e-8b41-4285-89d3-340408c8cd1c) - § 2 references coded [0.46% Coverage]

Reference 1 - 0.22% Coverage

R: They should continue it has helped us they try and continue.

Reference 2 - 0.24% Coverage

R: They should still give us and spread it to every part of Ghana.

[<Internals\\IDIs health volunteers\\IDI 36 year old Health volunteer-Tanziir>](file:///C:\Users\chatio\Desktop\Save%20in%20drive\studies\PK\SMC%20report\Final%20SMC%20report\Volunteers\Acceptability\1a01ef21-0554-43f3-acd3-340408de3ab4) - § 2 references coded [1.34% Coverage]

Reference 1 - 0.54% Coverage

R: If it gets to all places it would be good, because we have seen it benefits here, how it reduced the numbers at health facilities.

Reference 2 - 0.80% Coverage

R: What I will say is they should do well and let the drug continue to come and save the children. So what I will say is whatever they can do to sustain this program to go forward will be very good.

[<Internals\\IDIs health volunteers\\IDI 45 year old Health volunteer- Kolbugnuor>](file:///C:\Users\chatio\Desktop\Save%20in%20drive\studies\PK\SMC%20report\Final%20SMC%20report\Volunteers\Acceptability\db73d9f0-71e5-4376-86d3-340409449585) - § 1 reference coded [0.62% Coverage]

Reference 1 - 0.62% Coverage

Q. Should the program be introduced in other districts?

R. Yes, because it has helped us and i know will help them also

[<Internals\\IDIs health volunteers\\IDI 45 year old Health volunteer-Gbier>](file:///C:\Users\chatio\Desktop\Save%20in%20drive\studies\PK\SMC%20report\Final%20SMC%20report\Volunteers\Acceptability\dfa339b5-1827-441d-9bd3-340409553f66) - § 1 reference coded [0.42% Coverage]

Reference 1 - 0.42% Coverage

R. No. If only they can give us and still take to other districts

[<Internals\\IDIs health volunteers\\IDI 47 year old Health volunteer-Berwong>](file:///C:\Users\chatio\Desktop\Save%20in%20drive\studies\PK\SMC%20report\Final%20SMC%20report\Volunteers\Acceptability\6fdfd973-722e-4f1e-86d3-340409612512) - § 2 references coded [0.70% Coverage]

Reference 1 - 0.31% Coverage

R: They should continue because they agree to take it they should continue to bring it.

Reference 2 - 0.39% Coverage

R: Since we are all Ghanaian and need good health if they have the power they should extend it to every place.

[<Internals\\IDIs health volunteers\\IDI-50 year health volunteer-Zambo>](file:///C:\Users\chatio\Desktop\Save%20in%20drive\studies\PK\SMC%20report\Final%20SMC%20report\Volunteers\Acceptability\1c65b50f-05fe-4b67-b5d3-3eaa61dbf9c2) - § 1 reference coded [0.69% Coverage]

Reference 1 - 0.69% Coverage

R; Yes

Q; will you advice that they sent this medicine to other districts

R; Yes if only they will accept it
