# Supplementary material for: Community acceptability of Seasonal Malaria Chemoprevention of morbidity and mortality in young children: A qualitative study in the Upper West Region of Ghana
Source: PLoS One. 2019 May 17;14(5):e0216486. doi: 10.1371/journal.pone.0216486 (PMC6524792; doi:10.1371/journal.pone.0216486)
Supplement: S1 File — (ZIP) [file pone.0216486.s001.zip › Study data set-Nvivo coding/Knowledge on the rationale of the SMC.docx]

Knowledge on the rationale of the SMC drug

**IDIs with mothers**

[<Internals\\IDIs health workers\\IDIs mothers\\IDI 18 year old mother-Tanziir>](file:///C:\Users\chatio\Desktop\Save%20in%20drive\studies\PK\SMC%20report\Final%20SMC%20report\Mothers\Knowledge%20and%20adherence\d9286b71-0d0b-4b69-a3d3-30b1fce77d91) - § 1 reference coded [3.52% Coverage]

Reference 1 - 3.52% Coverage

Q: But what does it do to your child?

R: It has helped my child.

Q: It has helped your child in what way?

R: It stops diseases that used to worry him from worrying him.

[<Internals\\IDIs health workers\\IDIs mothers\\IDI 20 year old mother-Gbier>](file:///C:\Users\chatio\Desktop\Save%20in%20drive\studies\PK\SMC%20report\Final%20SMC%20report\Mothers\Knowledge%20and%20adherence\6d8a376a-063d-4b88-94d3-30b1fe15cc3a) - § 1 reference coded [0.80% Coverage]

Reference 1 - 0.80% Coverage

Q: What do the drugs they gave do for your child?

R: It protects the child from getting malaria?

[<Internals\\IDIs health workers\\IDIs mothers\\IDI 20 yearold mother-Berwong1>](file:///C:\Users\chatio\Desktop\Save%20in%20drive\studies\PK\SMC%20report\Final%20SMC%20report\Mothers\Knowledge%20and%20adherence\c8dc56f9-1da6-4c14-88d3-30b1fe3e4370) - § 1 reference coded [0.56% Coverage]

Reference 1 - 0.56% Coverage

R: The drug is given to prevent the child from been infected with malaria.

[<Internals\\IDIs health workers\\IDIs mothers\\IDI 26 year old mother-Newtown>](file:///C:\Users\chatio\Desktop\Save%20in%20drive\studies\PK\SMC%20report\Final%20SMC%20report\Mothers\Knowledge%20and%20adherence\d2cb5fdf-6b7b-4d12-b6d3-30b1fe4a2a75) - § 1 reference coded [1.27% Coverage]

Reference 1 - 1.27% Coverage

R. It is to protect the child from malaria.

[<Internals\\IDIs health workers\\IDIs mothers\\IDI 26 yearold mother-Eremon Tangzu (Autosaved)>](file:///C:\Users\chatio\Desktop\Save%20in%20drive\studies\PK\SMC%20report\Final%20SMC%20report\Mothers\Knowledge%20and%20adherence\dfa639bf-3760-46df-96d3-30b1fe5ad475) - § 1 reference coded [0.28% Coverage]

Reference 1 - 0.28% Coverage

R: I know that it protects children against malaria.

[<Internals\\IDIs health workers\\IDIs mothers\\IDI 27 year old mother-Newtown>](file:///C:\Users\chatio\Desktop\Save%20in%20drive\studies\PK\SMC%20report\Final%20SMC%20report\Mothers\Knowledge%20and%20adherence\6ada4bad-92df-4de8-93d3-30b1fe7503c8) - § 1 reference coded [0.48% Coverage]

Reference 1 - 0.48% Coverage

R. To prevent the child from getting malaria

[<Internals\\IDIs health workers\\IDIs mothers\\IDI 28 year old mother-Berwong>](file:///C:\Users\chatio\Desktop\Save%20in%20drive\studies\PK\SMC%20report\Final%20SMC%20report\Mothers\Knowledge%20and%20adherence\74cb6986-f6d6-4621-99d3-30b1fe80eacc) - § 2 references coded [3.55% Coverage]

Reference 1 - 2.87% Coverage

R: They normally give him to protect him from diseases like malaria, the malaria that worry the children. That is what I was saying previously when my child and I get to the hospital all times they normally say it is malaria, but they would give me the malaria drug to come home still day will not break but I go again and they tell me it is malaria but they will be given him blood and water but when they just gave us this drug this drug I will say it has help me when I gave birth to this child is about 8 months now send him to the hospital and they gave me drug till now he is not been sick again.

Reference 2 - 0.68% Coverage

Q: But I’m asking what they give the drug to do to the child?

R: That why I said concerning our disease.

Q: What disease?

R: The malaria.

[<Internals\\IDIs health workers\\IDIs mothers\\IDI 30 year old mother-Eremon Tangzu>](file:///C:\Users\chatio\Desktop\Save%20in%20drive\studies\PK\SMC%20report\Final%20SMC%20report\Mothers\Knowledge%20and%20adherence\99dc5250-fb67-4d4d-8ad3-30b1fea49eb2) - § 1 reference coded [5.02% Coverage]

Reference 1 - 5.02% Coverage

Q: What does this drug do to the child?

R: It makes the child healthy.

Q: I’m asking if they give the child the drug, what do they give it to do to him?

R: They gave to treat him of diseases.

[<Internals\\IDIs health workers\\IDIs mothers\\IDI 30 year old mother-Gbier>](file:///C:\Users\chatio\Desktop\Save%20in%20drive\studies\PK\SMC%20report\Final%20SMC%20report\Mothers\Knowledge%20and%20adherence\90c36595-6809-4c06-96d3-30b1fee4e4c2) - § 2 references coded [1.92% Coverage]

Reference 1 - 0.59% Coverage

R: They want to prevent my child from malaria infection.

Reference 2 - 1.33% Coverage

R: I think they saw that our children principal sickness is malaria and so they gave this drug to protect them from malaria

[<Internals\\IDIs health workers\\IDIs mothers\\IDI 30 year old mother-Kolbugnuor>](file:///C:\Users\chatio\Desktop\Save%20in%20drive\studies\PK\SMC%20report\Final%20SMC%20report\Mothers\Knowledge%20and%20adherence\452bf184-0cec-4af6-95d3-30b1feee696f) - § 1 reference coded [0.98% Coverage]

Reference 1 - 0.98% Coverage

Q. What is the benefits of these drugs to your child?

R. It help protect him from malaria

[<Internals\\IDIs health workers\\IDIs mothers\\IDI 30 year old mother-Newtown>](file:///C:\Users\chatio\Desktop\Save%20in%20drive\studies\PK\SMC%20report\Final%20SMC%20report\Mothers\Knowledge%20and%20adherence\1950e505-2542-4323-b9d3-30b1fef7ef36) - § 1 reference coded [1.90% Coverage]

Reference 1 - 1.90% Coverage

Q: Can you tell me what you know about the drug they gave to your children?

R: It is protect children against malaria.

[<Internals\\IDIs health workers\\IDIs mothers\\IDI 30 year old mother-Tuma>](file:///C:\Users\chatio\Desktop\Save%20in%20drive\studies\PK\SMC%20report\Final%20SMC%20report\Mothers\Knowledge%20and%20adherence\713306ab-8ca2-47cb-96d3-30b1feff1321) - § 1 reference coded [0.97% Coverage]

Reference 1 - 0.97% Coverage

Q: So, what does drug do to your child?

R: It protects him from malaria and let the child looks nice.

[<Internals\\IDIs health workers\\IDIs mothers\\IDI 31 year old mother-Eremon Tangzu>](file:///C:\Users\chatio\Desktop\Save%20in%20drive\studies\PK\SMC%20report\Final%20SMC%20report\Mothers\Knowledge%20and%20adherence\07b07256-641a-4fb8-9fd3-30b1ff147f2e) - § 1 reference coded [3.76% Coverage]

Reference 1 - 3.76% Coverage

Q: So if they give the child the drug that what does it do to the child?

R: We were told the child have to eat first before the drug is give. They said that, this will enable the drug to work effectively in preventing malaria in the child.

Q: Does it mean the child will not get malaria again?

R: He may get malaria but it won’t be serious like how it used to be.

Q: I want to again know, what they said the drug will do to the child?

R: They drug will kill the malaria parasite child’s body.

[<Internals\\IDIs health workers\\IDIs mothers\\IDI 31 year old mother-Kolbugnuor>](file:///C:\Users\chatio\Desktop\Save%20in%20drive\studies\PK\SMC%20report\Final%20SMC%20report\Mothers\Knowledge%20and%20adherence\e249c295-786b-44e2-96d3-30b1ff33714a) - § 1 reference coded [1.29% Coverage]

Reference 1 - 1.29% Coverage

Q; What do you know about this medicine?

R; The medicine is to protect the child against cold (malaria).

[<Internals\\IDIs health workers\\IDIs mothers\\IDI 34 year old mother-Tuma>](file:///C:\Users\chatio\Desktop\Save%20in%20drive\studies\PK\SMC%20report\Final%20SMC%20report\Mothers\Knowledge%20and%20adherence\49e33dc3-05d2-443e-b6d3-30b1ff71558e) - § 1 reference coded [1.62% Coverage]

Reference 1 - 1.62% Coverage

Q: So, when your child took the medicine what did it do to him?

R: Small, small sicknesses that the child was suffering with are all gone; he is now free roaming about.

[<Internals\\IDIs health workers\\IDIs mothers\\IDI 35 year old mother-Bagri>](file:///C:\Users\chatio\Desktop\Save%20in%20drive\studies\PK\SMC%20report\Final%20SMC%20report\Mothers\Knowledge%20and%20adherence\4d2676eb-d04a-4099-a3d3-30b1ff7f9d5c) - § 2 references coded [3.51% Coverage]

Reference 1 - 0.34% Coverage

R: It makes the malaria diseases to go down.

Reference 2 - 3.18% Coverage

R: This medicine has a lot of benefits to as, we don’t suffer getting it, we just sit in the house and get it, it is better than suffering to go to hospital.

Q: In what way?

R: When the child takes it, he looks well and eats plenty, so the body changes?

[<Internals\\IDIs health workers\\IDIs mothers\\IDI 35 year old mother-Tanziir>](file:///C:\Users\chatio\Desktop\Save%20in%20drive\studies\PK\SMC%20report\Final%20SMC%20report\Mothers\Knowledge%20and%20adherence\f4a9dcf4-f49d-44b0-9bd3-30b1ff9e8f9d) - § 2 references coded [3.42% Coverage]

Reference 1 - 1.75% Coverage

Q: what do you know concerning the drug they are giving to the children?

R: That they want to reduced malaria because it is killing our children, that is why the ‘’volunteer’’ brought the medicine to reduce it.

[<Internals\\IDIs health workers\\IDIs mothers\\IDI 37 year old mother-Kolbugnuor>](file:///C:\Users\chatio\Desktop\Save%20in%20drive\studies\PK\SMC%20report\Final%20SMC%20report\Mothers\Knowledge%20and%20adherence\e04a7c1d-5fa7-4576-8dd3-30b1ffd54e38) - § 2 references coded [6.79% Coverage]

Reference 1 - 3.42% Coverage

R; It is to protect the child from malaria and also to treat a child that is suffering from malaria. Since it protects the child against malaria, the child doesn`t fall sick and that makes the child strong in health.

Q; Do you have any other thing to add?

R; It also gives the child strength.

[<Internals\\IDIs health workers\\IDIs mothers\\IDI 50 year old mother-Ngman-gbil>](file:///C:\Users\chatio\Desktop\Save%20in%20drive\studies\PK\SMC%20report\Final%20SMC%20report\Mothers\Knowledge%20and%20adherence\2c4c0db0-dba2-4de3-bcd3-30b1ffdc73e0) - § 3 references coded [3.69% Coverage]

Reference 1 - 1.63% Coverage

R: I will say the medicine is very good; the children who took it, it protects them, they got ill two times and I went for medicine and still they got ill. Now I don’t see that again.

Reference 2 - 0.43% Coverage

R: It prevents malaria from affecting the child.

[<Internals\\IDIs health workers\\IDIs mothers\\IDI-23 year old mother-Berwong>](file:///C:\Users\chatio\Desktop\Save%20in%20drive\studies\PK\SMC%20report\Final%20SMC%20report\Mothers\Knowledge%20and%20adherence\1361ca8d-5b5d-4820-88d3-3eaa2e45ad3d) - § 3 references coded [3.04% Coverage]

Reference 1 - 1.59% Coverage

R: ok when they gave it us, I will say it is good, concerning malaria treatment.

Q: what has it done to show that it is good for malaria treatment?

R: ever since I started giving the child the drug she has never felt sick for doctors to say it is malaria so that she is given malaria treatment again that is why I said it is good.

**FGDs with fathers and mothers**

[<Internals\\FGDs\\FGD fathers with children under five-Bagri>](file:///C:\Users\chatio\Desktop\Save%20in%20drive\studies\PK\SMC%20report\Final%20SMC%20report\FGDs\Knowledge%20and%20adherence\554d2c4c-5aa1-43b0-afd3-2cbc7eb25cc8) - § 1 reference coded [1.95% Coverage]

Reference 1 - 1.95% Coverage

Q. What do you think the drug does to the children?

R6. It fights and kills the malaria parasites in the children.

R5. The drug protects the child from malaria even if the child is bitten by mosquitoes.

R8. The drug totally kills the malaria parasites in the children under five. I will plead you share these drugs to everybody not only children under five.

[<Internals\\FGDs\\FGD fathers with children under five-Tanziir>](file:///C:\Users\chatio\Desktop\Save%20in%20drive\studies\PK\SMC%20report\Final%20SMC%20report\FGDs\Knowledge%20and%20adherence\12582ff3-0c50-46c7-82d3-3403c9ad39ce) - § 2 references coded [1.56% Coverage]

Reference 1 - 0.48% Coverage

No.7

R: They said it is a malaria drug, so if you give it to child it can protect the child from getting malaria.

Reference 2 - 1.08% Coverage

No.4

R: The drug given to the children I have realised that they have good health how they use to have minor illnesses it is no more like that now.

Q: What do you think this drug does to the child?

R: The drug helps the child to be healthy and grow well.

[<Internals\\FGDs\\FGD fathers with children under five-Zambo>](file:///C:\Users\chatio\Desktop\Save%20in%20drive\studies\PK\SMC%20report\Final%20SMC%20report\FGDs\Knowledge%20and%20adherence\903102be-0a51-4b0e-93d3-3403caff3dbb) - § 1 reference coded [0.38% Coverage]

Reference 1 - 0.38% Coverage

R; It protects our children against malaria.

[<Internals\\FGDs\\FGD mothers with children under five-Bagri>](file:///C:\Users\chatio\Desktop\Save%20in%20drive\studies\PK\SMC%20report\Final%20SMC%20report\FGDs\Knowledge%20and%20adherence\a0552c32-4da9-42f3-87d3-2cbc7f4d15ae) - § 2 references coded [1.75% Coverage]

Reference 1 - 0.37% Coverage

Q: Can you tell me what the drug does to the child?

No.9

R: That it should protect the child from getting malaria.

Reference 2 - 1.38% Coverage

No.7

R: If the child is having the malaria virus the drug kills the virus in the child’s body making the child runs diarrhea but because we don’t know we rather say the drug has given the child a disease. If the child runs diarrhea and you take the child to hospital and they give him/her ORS it normally stop.

Q: So you are saying if the child is having the virus the drug kills the virus?

R: Yes it kills the virus in the stomach.

[<Internals\\FGDs\\FGD mothers with children under five-Tanziir>](file:///C:\Users\chatio\Desktop\Save%20in%20drive\studies\PK\SMC%20report\Final%20SMC%20report\FGDs\Knowledge%20and%20adherence\7933f35b-9705-4934-95d3-3403cb0fe604) - § 8 references coded [3.97% Coverage]

Reference 1 - 0.51% Coverage

No.8

R: When my child takes this drug he is always healthy he never took it and got weak he body stronger than when he did not take the drug.

Reference 2 - 0.17% Coverage

Q: Strong like what?

R: He is very energetic.

Reference 4 - 1.03% Coverage

No.7

R: For me I have realize that this drug you brought the drug helped the children very well, because when I was having one of my children anytime he is sick and I take him to hospital they always check and say it is a malaria, but when he just took this drug he never got ill at all.

Reference 5 - 0.52% Coverage

Q: Who has something to add to what it does to the child?

No.5

R: the drug is given strength to children and opening their knowledge for them.

Reference 6 - 0.64% Coverage

Q: what shows that the drug is opening their knowledge for them?

R: the way they used to behave when they did not take the drug and now they have taken the drug is not the same.

Reference 7 - 0.28% Coverage

R: the child becomes more active than when the child did not take the drug yet.

Reference 8 - 0.42% Coverage

No.6

R: when the children took this drug children who used to suffer convulsion now we are no more experiencing that.

[<Internals\\FGDs\\FGD mothers with children under five-Zambo>](file:///C:\Users\chatio\Desktop\Save%20in%20drive\studies\PK\SMC%20report\Final%20SMC%20report\FGDs\Knowledge%20and%20adherence\35ad761a-e06d-40cf-bcd3-3403cb27b45e) - § 6 references coded [2.25% Coverage]

Reference 1 - 0.18% Coverage

**No: 5**

R: They gave us the drug to prevent our children from malaria.

Reference 2 - 0.47% Coverage

**No: 6**

R: The drug they brought to us to give to the children it has helped me I used to go to hospital all the time but since I started collecting this drug I never went there again.

Reference 3 - 0.52% Coverage

**N**: 1

R: When this drug did not come yet, anytime I take my child to hospital they normally say it is malaria but when I took this drug till now I have never been to hospital, so I know it has helped me.

Reference 5 - 0.49% Coverage

No.3

R: Because if your child is having malaria it makes the child body warm always and it can cause the child to suffer “gyimeh” but because of the malaria drug we experience gyimeh again.

Reference 6 - 0.10% Coverage

**No.**4

R: To save children from malaria.

[<Internals\\FGDs\\FGD-fathers with children under five-Gbier>](file:///C:\Users\chatio\Desktop\Save%20in%20drive\studies\PK\SMC%20report\Final%20SMC%20report\FGDs\Knowledge%20and%20adherence\72a70c42-22e7-42d9-a8d3-3ea9fb2b287c) - § 4 references coded [3.71% Coverage]

Reference 1 - 0.88% Coverage

No. 2

R: Know that the drug helping us a lot.

Q: How is it helping you?

R: The way our children used to get sick of malaria all the time, now it is no more like that so it is helping us. I can give testimony of this drug. The day they brought the drug my child was already sick and they gave him the drug, so the following day he was still ill and we took him the hospital. It was the same drug they brought and we told them that he has already taken that drug so they returned it and brought a different one because he was having the malaria virus already. After he was better the SP & AQ drug was then given to him, he has not been sick till now.

Reference 4 - 0.22% Coverage

R: To prevent the child from getting malaria.

[<Internals\\FGDs\\FGD-mothers with children under five-Gbier>](file:///C:\Users\chatio\Desktop\Save%20in%20drive\studies\PK\SMC%20report\Final%20SMC%20report\FGDs\Knowledge%20and%20adherence\18f67d6c-f146-473c-83d3-3ea9fb47b8e4) - § 3 references coded [2.04% Coverage]

Reference 1 - 0.86% Coverage

R; The medicine helps the child to eat well. At first my child doesn`t eat much but now he eats a lot.

Reference 2 - 0.63% Coverage

R; The medicine is to protect our children from malaria and other diseases.

Reference 3 - 0.54% Coverage

R; It has protected our children from fever, diarrhea and polio.

**IDIs with health volunteers**

[<Internals\\IDIs health volunteers\\IDI 34 year old Health volunteer-Ngman-gbil>](file:///C:\Users\chatio\Desktop\Save%20in%20drive\studies\PK\SMC%20report\Final%20SMC%20report\Volunteers\Knowledge%20and%20adherence\9792d99e-569d-43a4-aad3-3404088aea56) - § 3 references coded [1.78% Coverage]

Reference 1 - 0.68% Coverage

R: Yeah, they said this medicine can protect our children from malaria, when we brought the medicine we realized it is true, if not in the past if you go to hospital every sick child is nothing but malaria.

Reference 2 - 0.85% Coverage

[<Internals\\IDIs health volunteers\\IDI 35 year old Health volunteer-Bagri>](file:///C:\Users\chatio\Desktop\Save%20in%20drive\studies\PK\SMC%20report\Final%20SMC%20report\Volunteers\Knowledge%20and%20adherence\570786a5-e7ba-4707-a3d3-340408a2b74c) - § 3 references coded [1.89% Coverage]

Reference 1 - 0.94% Coverage

R: They have realized that this region is having the highest rate of malaria infection that is why they brought it here to protects the child from malaria infection.

Q: What does the drug do to the child?

R: It protects the child from malaria infection.

Reference 2 - 0.51% Coverage

R: It also helps the child to eat. Some children were there they don’t eat but this drug has made them to eat well.

[<Internals\\IDIs health volunteers\\IDI 35 year old Health volunteer-Newtown>](file:///C:\Users\chatio\Desktop\Save%20in%20drive\studies\PK\SMC%20report\Final%20SMC%20report\Volunteers\Knowledge%20and%20adherence\e62ddbb9-5b7b-4af3-aed3-340408b8247e) - § 3 references coded [3.23% Coverage]

Reference 1 - 0.84% Coverage

Q. What is your knowledge on the SMC program in this district?

R. What i know is that it has helped reduced malaria cases

Reference 2 - 0.85% Coverage

Q. Why did they bring the program to this district?

R. They said we here are suffering from malaria and on an creasing rate

[<Internals\\IDIs health volunteers\\IDI 35 year old Health volunteer-Tuma>](file:///C:\Users\chatio\Desktop\Save%20in%20drive\studies\PK\SMC%20report\Final%20SMC%20report\Volunteers\Knowledge%20and%20adherence\ec8b1f3e-8b41-4285-89d3-340408c8cd1c) - § 4 references coded [3.10% Coverage]

Reference 1 - 1.42% Coverage

R: They said up north here that the malaria disease infecting rate is high especially among children who are not up to five years that is why they said they will do something and send medicine to this place so that they can give to the children for three years and they see what this disease will look like whether it will reduce here or it will still be like that. That is why they sent the drug.

Reference 3 - 0.47% Coverage

Q: Why should they give this drug to the child?

R: They normally give this drug to the child to be protected from getting malaria.

[<Internals\\IDIs health volunteers\\IDI 36 year old Health volunteer-Tanziir>](file:///C:\Users\chatio\Desktop\Save%20in%20drive\studies\PK\SMC%20report\Final%20SMC%20report\Volunteers\Knowledge%20and%20adherence\1a01ef21-0554-43f3-acd3-340408de3ab4) - § 4 references coded [3.67% Coverage]

Reference 1 - 1.57% Coverage

R: When they brought the drug they called and informed as how the drug worked they said the two drugs go together SP &AQ they also said someone is there when he/she takes this malaria drug the person can get rashes so we should not be afraid if we see that, so when the children took this drug we realize that the drug is good now if you are going anywhere people are asking for the drug.

[<Internals\\IDIs health volunteers\\IDI 45 year old Health volunteer- Kolbugnuor>](file:///C:\Users\chatio\Desktop\Save%20in%20drive\studies\PK\SMC%20report\Final%20SMC%20report\Volunteers\Knowledge%20and%20adherence\db73d9f0-71e5-4376-86d3-340409449585) - § 4 references coded [3.27% Coverage]

Reference 1 - 0.97% Coverage

R. They said children from three months to five years in these communities are mostly affected by malaria so the government saw it prudent to brig these drugs to protect them from malaria.

[<Internals\\IDIs health volunteers\\IDI 45 year old Health volunteer-Gbier>](file:///C:\Users\chatio\Desktop\Save%20in%20drive\studies\PK\SMC%20report\Final%20SMC%20report\Volunteers\Knowledge%20and%20adherence\dfa339b5-1827-441d-9bd3-340409553f66) - § 2 references coded [2.19% Coverage]

Reference 1 - 0.96% Coverage

R. I know they give drugs to children to protect them from malaria because a lot of malaria cases use to be recorded in our various health facilities

[<Internals\\IDIs health volunteers\\IDI 47 year old Health volunteer-Berwong>](file:///C:\Users\chatio\Desktop\Save%20in%20drive\studies\PK\SMC%20report\Final%20SMC%20report\Volunteers\Knowledge%20and%20adherence\6fdfd973-722e-4f1e-86d3-340409612512) - § 3 references coded [2.06% Coverage]

Reference 1 - 0.93% Coverage

R: Ok it was the mosquitoes that worry the children so we were also blowing the alarm at the hospital and when our leaders call us for a meeting we also blow the alarm there so they also inform the NGOs; so I think all that contributed to the coming of this drug.

Q: So, I ask why they should give the drug to the child.

R: It is for health reasons. The child should not be sick of malaria.

[<Internals\\IDIs health volunteers\\IDI-50 year health volunteer-Zambo>](file:///C:\Users\chatio\Desktop\Save%20in%20drive\studies\PK\SMC%20report\Final%20SMC%20report\Volunteers\Knowledge%20and%20adherence\1c65b50f-05fe-4b67-b5d3-3eaa61dbf9c2) - § 2 references coded [2.72% Coverage]

Reference 1 - 1.04% Coverage

R; To me it is of great help to us because our children use to suffer from malaria a lot and am urging that the health service should continue to bring this medicine

Reference 2 - 1.68% Coverage

R; Is because of malaria that they brought this medicine because we use to suffer from malaria alot

Q; Why do they give this medicine to the children

R; Children are the most affected in terms of malaria infection that is why they give this medicine to the children.
