# Supplementary material for: Community acceptability of Seasonal Malaria Chemoprevention of morbidity and mortality in young children: A qualitative study in the Upper West Region of Ghana
Source: PLoS One. 2019 May 17;14(5):e0216486. doi: 10.1371/journal.pone.0216486 (PMC6524792; doi:10.1371/journal.pone.0216486)
Supplement: S1 File — (ZIP) [file pone.0216486.s001.zip › Study data set-Nvivo coding/Patients adherence.docx]

**Patients’ adherence and completion of dosages**

**IDIs with mothers**

[<Internals\\IDIs health workers\\IDIs mothers\\IDI 18 year old mother-Tanziir>](file:///C:\Users\chatio\Desktop\Save%20in%20drive\studies\PK\SMC%20report\Final%20SMC%20report\Mothers\Knowledge%20and%20adherence\d9286b71-0d0b-4b69-a3d3-30b1fce77d91) - § 1 reference coded [2.11% Coverage]

Reference 1 - 2.11% Coverage

Q: So, he has taken them in four days, has he taken them without jumping a day?

R: Yes

Q: How many times have you taken the drug for your child?

R: Three times because I was not there when they started giving the drug to the children.

[<Internals\\IDIs health workers\\IDIs mothers\\IDI 20 year old mother-Gbier>](file:///C:\Users\chatio\Desktop\Save%20in%20drive\studies\PK\SMC%20report\Final%20SMC%20report\Mothers\Knowledge%20and%20adherence\6d8a376a-063d-4b88-94d3-30b1fe15cc3a) - § 1 reference coded [4.57% Coverage]

Reference 1 - 4.57% Coverage

Q: How many times did your child benefited (received) these drugs?

R: He received it twice.

Q: Why did he receive it twice? I think it is four times and why did he received it two times?

R: I was not here in the beginning that is why.

Q: When they came and met the child did he receive all?

R: Yes. He received all for the first time and then they brought the second batch for him.

Q: Did you give the drugs according to the prescription? OR Have you forgotten someday and could not give him the drugs?

R: Yes, i did it according to the prescription.

[<Internals\\IDIs health workers\\IDIs mothers\\IDI 20 yearold mother-Berwong1>](file:///C:\Users\chatio\Desktop\Save%20in%20drive\studies\PK\SMC%20report\Final%20SMC%20report\Mothers\Knowledge%20and%20adherence\c8dc56f9-1da6-4c14-88d3-30b1fe3e4370) - § 1 reference coded [1.46% Coverage]

Reference 1 - 1.46% Coverage

Q: How many times has your child taken this medicine?

R: Four times.

Q: Have you administered the medicine the way you have explained?

R: Yes.

[<Internals\\IDIs health workers\\IDIs mothers\\IDI 26 year old mother-Newtown>](file:///C:\Users\chatio\Desktop\Save%20in%20drive\studies\PK\SMC%20report\Final%20SMC%20report\Mothers\Knowledge%20and%20adherence\d2cb5fdf-6b7b-4d12-b6d3-30b1fe4a2a75) - § 2 references coded [3.16% Coverage]

Reference 1 - 2.57% Coverage

Q. Have your child completed this dosage?

R. Yes

Q. How many times did your child take the drugs?

R. Three times

Q. To the best of my knowledge, I think it is suppose to be four times, so why didn’t your child take the fourth dose?

R. I was not around for the forth dose.

[<Internals\\IDIs health workers\\IDIs mothers\\IDI 26 yearold mother-Eremon Tangzu (Autosaved)>](file:///C:\Users\chatio\Desktop\Save%20in%20drive\studies\PK\SMC%20report\Final%20SMC%20report\Mothers\Knowledge%20and%20adherence\dfa639bf-3760-46df-96d3-30b1fe5ad475) - § 2 references coded [2.80% Coverage]

Reference 1 - 2.50% Coverage

Q: How many times did your child receive the drug?

R: four times.

Q: So the way you described how the child is supposed to take the drug, is that how you give him?

R: Yes.

Q: Didn’t you forget to give him someday maybe because of work and gave to him later?

R: Because the last day we were having funeral, so I forgot to give it to him. The following day I asked the guy who was here and he said since he could not take it the previous day that he can’t take it again

Reference 2 - 0.31% Coverage

Q: So is only the fourth time that he missed a day.

R: Yes.

[<Internals\\IDIs health workers\\IDIs mothers\\IDI 27 year old mother-Newtown>](file:///C:\Users\chatio\Desktop\Save%20in%20drive\studies\PK\SMC%20report\Final%20SMC%20report\Mothers\Knowledge%20and%20adherence\6ada4bad-92df-4de8-93d3-30b1fe7503c8) - § 2 references coded [2.80% Coverage]

Reference 1 - 1.02% Coverage

Q. Did your children take all the drugs from the beginning to the end of the intervention?

R. Yes

Reference 2 - 1.78% Coverage

Q. Did your children follow exactly the times that you mentioned during the program or you forgot and mixed up the times?

R. That is how I gave the drugs to my children.

[<Internals\\IDIs health workers\\IDIs mothers\\IDI 28 year old mother-Berwong>](file:///C:\Users\chatio\Desktop\Save%20in%20drive\studies\PK\SMC%20report\Final%20SMC%20report\Mothers\Knowledge%20and%20adherence\74cb6986-f6d6-4621-99d3-30b1fe80eacc) - § 1 reference coded [1.24% Coverage]

Reference 1 - 1.24% Coverage

Q: He didn’t miss a day?

R: No.

Q: How you describe the way they asked you to administer this drug is there someday you forgot to give the way they asked you to give him?

R: No, the way they directed me to be given to him that is the way I normally give him.

[<Internals\\IDIs health workers\\IDIs mothers\\IDI 30 year old mother-BagriE>](file:///C:\Users\chatio\Desktop\Save%20in%20drive\studies\PK\SMC%20report\Final%20SMC%20report\Mothers\Knowledge%20and%20adherence\f4681a8b-1b78-4cab-add3-30b1fe8f3281) - § 1 reference coded [3.24% Coverage]

Reference 1 - 3.24% Coverage

Q. How many time has your child received this drug?

R. It was about three times.

Q. Has your child taken all the dosages from the beginning to end?

R. Yes

[<Internals\\IDIs health workers\\IDIs mothers\\IDI 30 year old mother-Eremon Tangzu>](file:///C:\Users\chatio\Desktop\Save%20in%20drive\studies\PK\SMC%20report\Final%20SMC%20report\Mothers\Knowledge%20and%20adherence\99dc5250-fb67-4d4d-8ad3-30b1fea49eb2) - § 1 reference coded [2.51% Coverage]

Reference 1 - 2.51% Coverage

Q: How many times did you give the child the drug?

R: Four rounds.

Q: In these four rounds, have they ever brought it to be given the three days and you give less than the three days or more than the three times, or you are suppose to give in the morning and you rather give in the evening because you have forgotten or you gave him exactly how you were told?

R: I give to him every morning for the three days.

[<Internals\\IDIs health workers\\IDIs mothers\\IDI 30 year old mother-Gbier>](file:///C:\Users\chatio\Desktop\Save%20in%20drive\studies\PK\SMC%20report\Final%20SMC%20report\Mothers\Knowledge%20and%20adherence\90c36595-6809-4c06-96d3-30b1fee4e4c2) - § 1 reference coded [2.98% Coverage]

Reference 1 - 2.98% Coverage

Q: How many times did your children receive these drugs?

R: Two times

Q: I mean why did your child receive it twice instead of four times?

R: I was not around because my mother was sick and was admitted elsewhere.

[<Internals\\IDIs health workers\\IDIs mothers\\IDI 30 year old mother-Kolbugnuor>](file:///C:\Users\chatio\Desktop\Save%20in%20drive\studies\PK\SMC%20report\Final%20SMC%20report\Mothers\Knowledge%20and%20adherence\452bf184-0cec-4af6-95d3-30b1feee696f) - § 1 reference coded [3.08% Coverage]

Reference 1 - 3.08% Coverage

Q. How many times did your child take the drugs?

R. Three times

Q. So did your child take all the drugs from the beginning of the program to the end?

R. Yes, he has not missed any of them

[<Internals\\IDIs health workers\\IDIs mothers\\IDI 30 year old mother-Newtown>](file:///C:\Users\chatio\Desktop\Save%20in%20drive\studies\PK\SMC%20report\Final%20SMC%20report\Mothers\Knowledge%20and%20adherence\1950e505-2542-4323-b9d3-30b1fef7ef36) - § 2 references coded [2.06% Coverage]

Reference 1 - 1.04% Coverage

Q: Have you given your child this medicine as prescribed?

R: Yes.

Q: How many times has your child taken the medicine?

R: Three.

[<Internals\\IDIs health workers\\IDIs mothers\\IDI 31 year old mother-Eremon Tangzu>](file:///C:\Users\chatio\Desktop\Save%20in%20drive\studies\PK\SMC%20report\Final%20SMC%20report\Mothers\Knowledge%20and%20adherence\07b07256-641a-4fb8-9fd3-30b1ff147f2e) - § 1 reference coded [1.26% Coverage]

Reference 1 - 1.26% Coverage

Q: How many times did your child took this drug?

R: Three times.

Q: When they started to the end did he took all the rounds?

R: Yes.

[<Internals\\IDIs health workers\\IDIs mothers\\IDI 31 year old mother-Kolbugnuor>](file:///C:\Users\chatio\Desktop\Save%20in%20drive\studies\PK\SMC%20report\Final%20SMC%20report\Mothers\Knowledge%20and%20adherence\e249c295-786b-44e2-96d3-30b1ff33714a) - § 1 reference coded [5.08% Coverage]

Reference 1 - 5.08% Coverage

Q; How many times has your child taken this medicine?

R; Three times.

Q; Since they started giving the medicine has your child skipped any month without taking the medicine.

R; No, he has never skipped.

Q; In the process of administering the medicine to your child, have you ever forgotten or skip a day without giving the medicine to the child?

R; No, I have never forgotten to administer the medicine to my child.

[<Internals\\IDIs health workers\\IDIs mothers\\IDI 31 year old mother-Tuma>](file:///C:\Users\chatio\Desktop\Save%20in%20drive\studies\PK\SMC%20report\Final%20SMC%20report\Mothers\Knowledge%20and%20adherence\70adc82b-bbde-47e1-8fd3-31c514093d39) - § 1 reference coded [2.97% Coverage]

Reference 1 - 2.97% Coverage

Q: This medicine how many times has your child taken it?

R: It is like four times.

Q: So, the child has taken the medicine all the four times?

R: Yes.

Q: So your child all the four times you have taken the medicine, for the three days that you have to administer the medicine, you never jump a day?

R: Yes he never jumped a day. He took all the medicine throughout.

[<Internals\\IDIs health workers\\IDIs mothers\\IDI 32 year old mother-Gbier>](file:///C:\Users\chatio\Desktop\Save%20in%20drive\studies\PK\SMC%20report\Final%20SMC%20report\Mothers\Knowledge%20and%20adherence\169607a2-22bc-48ff-b4d3-31cc71f08747) - § 1 reference coded [2.37% Coverage]

Reference 1 - 2.37% Coverage

Q; how many times has your child taken this drugs?

R: four times

Q: have you followed the exact prescriptions?

R: yes.Even the lady in charge did a follow up the next day to see whether I have given my child the drugs. She asked me personally.

[<Internals\\IDIs health workers\\IDIs mothers\\IDI 34 year old mother-Tuma>](file:///C:\Users\chatio\Desktop\Save%20in%20drive\studies\PK\SMC%20report\Final%20SMC%20report\Mothers\Knowledge%20and%20adherence\49e33dc3-05d2-443e-b6d3-30b1ff71558e) - § 1 reference coded [1.15% Coverage]

Reference 1 - 1.15% Coverage

Q: Your child when they started to the end has he taken all them?

R: Yes

Q: How many times?

R: Three times

Q: But did he take all?

R: Yes, he took everything.

[<Internals\\IDIs health workers\\IDIs mothers\\IDI 35 year old mother-Tanziir>](file:///C:\Users\chatio\Desktop\Save%20in%20drive\studies\PK\SMC%20report\Final%20SMC%20report\Mothers\Knowledge%20and%20adherence\f4a9dcf4-f49d-44b0-9bd3-30b1ff9e8f9d) - § 1 reference coded [1.93% Coverage]

Reference 1 - 1.93% Coverage

R: No, the number of days they teach me to give him the same of days I normally take, the time they give me is the same time I normally give him too.

Q: So you never missed a day you never missed a time?

R: Yes

[<Internals\\IDIs health workers\\IDIs mothers\\IDI 36 year old mother-Bagri>](file:///C:\Users\chatio\Desktop\Save%20in%20drive\studies\PK\SMC%20report\Final%20SMC%20report\Mothers\Knowledge%20and%20adherence\8687b6b6-f1c5-444b-acd3-30b1ffbfe296) - § 3 references coded [2.73% Coverage]

Reference 1 - 0.46% Coverage

Q: So, has your child taken all the drugs?

R: Yes, everything.

Reference 2 - 1.77% Coverage

R: She took when she was 3months old and when she was 4months

Q: So, how many times has she taken?

R: Two times.

Q: What about the other two months she has not taken, what was the reason?

R: At that time she was not qualify to take drugs.

Reference 3 - 0.49% Coverage

Q: What makes her not qualify?

R: They said until she is 3 months.

[<Internals\\IDIs health workers\\IDIs mothers\\IDI 36 year old mother-Ngman-gbil>](file:///C:\Users\chatio\Desktop\Save%20in%20drive\studies\PK\SMC%20report\Final%20SMC%20report\Mothers\Knowledge%20and%20adherence\d09e840c-5e47-4690-a9d3-30b1ffcbc9dc) - § 1 reference coded [1.20% Coverage]

Reference 1 - 1.20% Coverage

R: I’ve taken two times.

Q: You’ve taken two times, why two times?

R: The time they started I wasn’t there but I came and met it.

**FGDs with fathers and mothers**

[<Internals\\FGDs\\FGD fathers with children under five-Bagri>](file:///C:\Users\chatio\Desktop\Save%20in%20drive\studies\PK\SMC%20report\Final%20SMC%20report\FGDs\Knowledge%20and%20adherence\554d2c4c-5aa1-43b0-afd3-2cbc7eb25cc8) - § 1 reference coded [3.82% Coverage]

Reference 1 - 3.82% Coverage

Q. I want to ask each of you one after the other, Have your child completed the entire dosage?

R1. My has taken but whenever they are coming to give him the drug, am not always home but my wife.

R2. Yest, my child has completed the entire dosage.

R3. Since they started, my child has always taken it and hence has completed the entire dosage

R4. Yes my child has completed the entire dosage

R5. As for me, i traveled in the process so i cant best tell whether my child has completed the entire dosage

R6. Yes my child has never missed any dosage

R7. I am not always home, so i cant confidently say because it is my wife that can best know

R8. My child has taken all dosages

R9. My child has not missed

[<Internals\\FGDs\\FGD fathers with children under five-Tanziir>](file:///C:\Users\chatio\Desktop\Save%20in%20drive\studies\PK\SMC%20report\Final%20SMC%20report\FGDs\Knowledge%20and%20adherence\12582ff3-0c50-46c7-82d3-3403c9ad39ce) - § 7 references coded [1.64% Coverage]

Reference 1 - 0.16% Coverage

No.1

R: My child has taken everything.

Reference 2 - 0.24% Coverage

Q: How many times did he take this drug?

R: Four times.

Reference 3 - 0.21% Coverage

Q: He did not jump any?

R: No he took everything.

Reference 4 - 0.48% Coverage

R: He took it four times.

No.3

R: It is the same as what No.2 said.

No.4

R: That is it he took it four times.

Reference 5 - 0.13% Coverage

No.5

R: He took it four times.

Reference 6 - 0.21% Coverage

No.6

R: It is four times it is even on the wall.

Reference 7 - 0.22% Coverage

R: My child took it four times.

No.8

R: Four times.

[<Internals\\FGDs\\FGD mothers with children under five-Bagri>](file:///C:\Users\chatio\Desktop\Save%20in%20drive\studies\PK\SMC%20report\Final%20SMC%20report\FGDs\Knowledge%20and%20adherence\a0552c32-4da9-42f3-87d3-2cbc7f4d15ae) - § 1 reference coded [10.33% Coverage]

Reference 1 - 10.33% Coverage

Q: I will start with No.1, No.1 how many times did your child take the drug?

No.1

R: Three times.

Q: From the beginning to the end has he taken everything?

R: Yes.

Q: And you said three times? I you sure you never jumped?

R: Yes.

Q: But on the daily bases there is not a day you forgot and jumped?

R: No I never forgot. It is four times I forgot.

Q: Is it four times you collected?

R: Yes.

Q: No.2, how many times did your child collect the drug?

No.2

R: Four times.

Q: From the start to the end he collected everything?

R: Yes.

Q: But let take how you supposed to give the child the drug, do you give him how you supposed to give him? Is there not a day you forgot to give him?

R: No, I give him rightly.

Q: No.3 has she taken everything or some month she could not collect?

No.3

R: No she collected everything.

Q: Has she collected for all the months?

R: Yes.

Q: But concerning giving the drug to the child, have you her everything or because work you forgot to give her some day?

R: No I have giving her everything I’ve never miss a day.

Q: If you give the drug to the child in the morning the following day is it after you give her again?

R: No, if give to her morning I maintain giving in the morning for the three days.

Q: No. 4, how many times did you child collect the drug?

No.4

R: Three times, the first round they brought he was admitted at the hospital so he could not collect.

Q: But for the three rounds have you given him all or you forgot to give him someday because you forgot?

R: I gave him everything.

No.5

R: My child took the drug four times. There are four always tablets, I normally give the white and yellow tablets together for the first day and the second day I give him one and the following day I give one to complete it.

Q: You haven’t forgotten to give the drug to the child someday?

R: No I didn’t miss any day.

No.6

R: My child took it four times. The first day when they brought it you have to pick the white and the yellow for the child the first day, you normally put the yellow in the water to dissolve and you give to the child to and also put the white to also dissolve and you give to the child to, day 2 you dissolve for the child and they day 3 you dissolve one for him to. They are always four tablets in the in the sachet

Q: You haven’t forgotten to give the drug to the child someday?

R: Yes.

Q: No.7 how many times did your child take the drug?

No.7

R: Three times.

Q: From the beginning to the end did he collect everything?

R: He didn’t, he was admitted at the hospital so he couldn’t take the first round. This baby I’m holding was not also up to 3months then.

Q: So many times did the baby collect?

R: Three times to.

Q: But were you able to give the children how they suppose to take the drug or you miss someday?

R: No, I give to them how they are supposed to take it I didn’t miss a day. I was given it to the elderly one at 7pm after meals every day and the baby in the morning.

Q: No.8, how many times did your child take the drug?

No.8

R: Four times.

Q: And were you able to give him how were told to do it?

R: Yes, I always him those two the following days one, one till the end.

Q: No.9, how many times did he collect?

No.9

R: Four times the same way.

[<Internals\\FGDs\\FGD mothers with children under five-Tanziir>](file:///C:\Users\chatio\Desktop\Save%20in%20drive\studies\PK\SMC%20report\Final%20SMC%20report\FGDs\Knowledge%20and%20adherence\7933f35b-9705-4934-95d3-3403cb0fe604) - § 5 references coded [2.16% Coverage]

Reference 1 - 0.23% Coverage

R: yes.

Q: how many times did he take the drug?

R: four times.

Reference 2 - 0.54% Coverage

R: the way her child takes the malaria drug that is how my child also takes it.

Q: how many times did your child takes the drug?

R: four times.

No.3

Reference 3 - 0.42% Coverage

R: the child took it four times.

Q: the three days that he is supposed to take it he didn’t jump any of them?

R: no.

Reference 4 - 0.82% Coverage

Q: then it is four times or times?

R: yes it is four times and I said three times.

Q: No.7

R: the drug they brought, it is four times that they gave to us. They gave me in the afternoon so the child takes 1 o’clock every day.

Reference 5 - 0.14% Coverage

R: four months.

Q: No.9

R: four times.

[<Internals\\FGDs\\FGD mothers with children under five-Zambo>](file:///C:\Users\chatio\Desktop\Save%20in%20drive\studies\PK\SMC%20report\Final%20SMC%20report\FGDs\Knowledge%20and%20adherence\35ad761a-e06d-40cf-bcd3-3403cb27b45e) - § 2 references coded [0.32% Coverage]

Reference 1 - 0.13% Coverage

No.7

R: My child collected for all the four times.

Reference 2 - 0.19% Coverage

Q: But you didn’t forget to give him someday?

R: No he took everything.

[<Internals\\FGDs\\FGD-fathers with children under five-Gbier>](file:///C:\Users\chatio\Desktop\Save%20in%20drive\studies\PK\SMC%20report\Final%20SMC%20report\FGDs\Knowledge%20and%20adherence\72a70c42-22e7-42d9-a8d3-3ea9fb2b287c) - § 5 references coded [2.46% Coverage]

Reference 1 - 0.47% Coverage

R: My child took it two times.

Q: Why two times?

R: He wasn’t due to collect when they started.

Reference 2 - 0.35% Coverage

Q: But did he collect everything from the beginning to the end?

R: Yes.

Reference 3 - 0.61% Coverage

R: It was the second time that my child was given the drug.

Q: Why?

R: He was not due to collect the drug when they started.

Reference 4 - 0.48% Coverage

R: For my child when they started he took everything to the end.

Q: How many times?

R: Four times.

Reference 5 - 0.56% Coverage

No.10

R: He took everything from the start to the end.

No.11

R: When they started to the end he took everything.

[<Internals\\FGDs\\FGD-mothers with children under five-Gbier>](file:///C:\Users\chatio\Desktop\Save%20in%20drive\studies\PK\SMC%20report\Final%20SMC%20report\FGDs\Knowledge%20and%20adherence\18f67d6c-f146-473c-83d3-3ea9fb47b8e4) - § 2 references coded [1.38% Coverage]

Reference 1 - 1.14% Coverage

R; My child tookit three times.

Q; How many months has your child taken the medicine?

R; Four months.

No. 3

R; Three months.

Reference 2 - 0.24% Coverage

No. 4

R; Four months.

[<Internals\\FGDs\\FGD-fathers with children under five-Gbier>](file:///C:\Users\chatio\Desktop\Save%20in%20drive\studies\PK\SMC%20report\Final%20SMC%20report\FGDs\Knowledge%20and%20adherence\72a70c42-22e7-42d9-a8d3-3ea9fb2b287c) - § 2 references coded [1.08% Coverage]

Reference 1 - 0.47% Coverage

R: My child took it two times.

Q: Why two times?

R: He wasn’t due to collect when they started.

Reference 2 - 0.61% Coverage

R: It was the second time that my child was given the drug.

Q: Why?

R: He was not due to collect the drug when they started.

**IDIs with health volunteers**

[<Internals\\IDIs health volunteers\\IDI 34 year old Health volunteer-Ngman-gbil>](file:///C:\Users\chatio\Desktop\Save%20in%20drive\studies\PK\SMC%20report\Final%20SMC%20report\Volunteers\Knowledge%20and%20adherence\9792d99e-569d-43a4-aad3-3404088aea56) - § 2 references coded [1.73% Coverage]

Reference 1 - 0.59% Coverage

R: Yes, when you give them the drug the following day you have to start from where you started the previous day and go round and find out whether have given the drug to the child.

Reference 2 - 1.14% Coverage

R: That is what I’m saying you the volunteer also go if I go today I know the time I will tell the mother tomorrow at this time if am not able to come give the drug to the child. So when you go later you can ask whether she given it to the child if she says yes you ask for the one left if she brings it you know that she has given it to the child.

[<Internals\\IDIs health volunteers\\IDI 35 year old Health volunteer-Bagri>](file:///C:\Users\chatio\Desktop\Save%20in%20drive\studies\PK\SMC%20report\Final%20SMC%20report\Volunteers\Knowledge%20and%20adherence\570786a5-e7ba-4707-a3d3-340408a2b74c) - § 4 references coded [4.67% Coverage]

Reference 1 - 0.74% Coverage

R: In our community here mothers are controversial so we give the drug to the children by ourselves; if not some can collect and keep it they won’t give to the child.

Reference 2 - 2.38% Coverage

R: It is because they are controversial if you give the drug to some of them they won’t give to the child so for us we normally give the drug to the child by ourselves and all the three days we normally go round and check, some children who are of the size my child if we go and give the child for day-one we always give the mother the two tablets to keep, the following morning they are the people we normally go round and check first whether they have giving it to the children or not before we then go and continue with the others.

Reference 3 - 1.45% Coverage

R: It is not that they don’t want to give it to the children, that is how we were trained, they worked with them and they know who they are, one can go to hospital and collect drugs and come and keep she won’t be serious and give it to the child to get cured, so it happened to be our work that is why we were also doing that.

Reference 4 - 0.09% Coverage

R: They accepted it.

[<Internals\\IDIs health volunteers\\IDI 35 year old Health volunteer-Newtown>](file:///C:\Users\chatio\Desktop\Save%20in%20drive\studies\PK\SMC%20report\Final%20SMC%20report\Volunteers\Knowledge%20and%20adherence\e62ddbb9-5b7b-4af3-aed3-340408b8247e) - § 1 reference coded [0.91% Coverage]

Reference 1 - 0.91% Coverage

Q. Do mothers usually accept this drug for their children?

R. As i said earlier, only few try rejecting because of the side effects

[<Internals\\IDIs health volunteers\\IDI 35 year old Health volunteer-Tuma>](file:///C:\Users\chatio\Desktop\Save%20in%20drive\studies\PK\SMC%20report\Final%20SMC%20report\Volunteers\Knowledge%20and%20adherence\ec8b1f3e-8b41-4285-89d3-340408c8cd1c) - § 3 references coded [1.84% Coverage]

Reference 1 - 0.99% Coverage

R: For that we give it to the children ourselves.

Q: But do they agree and give them?

R: Yes they agree, some are saying their children are difficult that they normally collect and the children do not agree to take it, but the child takes it if we are giving it to the child.

Reference 2 - 0.65% Coverage

Q: What makes it difficult for a child to take this drug?

R: Some children are there they don’t take drug, some take the liquid one but if it is the tablets if they take they vomit.

Reference 3 - 0.20% Coverage

[<Internals\\IDIs health volunteers\\IDI 36 year old Health volunteer-Tanziir>](file:///C:\Users\chatio\Desktop\Save%20in%20drive\studies\PK\SMC%20report\Final%20SMC%20report\Volunteers\Knowledge%20and%20adherence\1a01ef21-0554-43f3-acd3-340408de3ab4) - § 1 reference coded [1.19% Coverage]

Reference 1 - 1.19% Coverage

Q: But you said some collect the drug and just kept it.

R: Yes, that was the beginning some say their children do not agree to take it but when we force them to take it for two times, when we are going and they see us they normally come to us by themselves they know are bringing the drug again.

[<Internals\\IDIs health volunteers\\IDI 45 year old Health volunteer- Kolbugnuor>](file:///C:\Users\chatio\Desktop\Save%20in%20drive\studies\PK\SMC%20report\Final%20SMC%20report\Volunteers\Knowledge%20and%20adherence\db73d9f0-71e5-4376-86d3-340409449585) - § 3 references coded [2.58% Coverage]

Reference 1 - 0.48% Coverage

Q. What of the adherence rate?

R. We do monitoring to ensure that the children take the drugs

Reference 2 - 1.37% Coverage

Q. Left unto the mothers, do you thing they will give the drugs to the children or not?

R. Some women are very difficult; if you give the drug to them to be given some will not. That is why we usually do monitoring to ensure that the drugs are given to the children.

Reference 3 - 0.73% Coverage

Q. What reason is behind some mothers not ready to give the drugs if not monitored?

R. Some mothers say they have forgotten when it is time.

[<Internals\\IDIs health volunteers\\IDI 45 year old Health volunteer-Gbier>](file:///C:\Users\chatio\Desktop\Save%20in%20drive\studies\PK\SMC%20report\Final%20SMC%20report\Volunteers\Knowledge%20and%20adherence\dfa339b5-1827-441d-9bd3-340409553f66) - § 2 references coded [2.73% Coverage]

Reference 1 - 2.46% Coverage

Q. Do mothers give the right dosage to their children?

R. Yes. We the volunteers give the drugs. We gave to the children on the first and second round. It was on the third round that the mothers themselves complaint that we are tired , so we gave it to them and still went round to monitor. Sometimes the children do not allow their mothers to give the drugs so we have to intervene. Some of them complain that the drugs are bitter

[<Internals\\IDIs health volunteers\\IDI 47 year old Health volunteer-Berwong>](file:///C:\Users\chatio\Desktop\Save%20in%20drive\studies\PK\SMC%20report\Final%20SMC%20report\Volunteers\Knowledge%20and%20adherence\6fdfd973-722e-4f1e-86d3-340409612512) - § 3 references coded [0.97% Coverage]

Reference 1 - 0.07% Coverage

R: Yes they agreed.

Reference 2 - 0.12% Coverage

days?

R: No they didn’t jump days.

Reference 3 - 0.78% Coverage

R: They can administer it but not all can do that if you don’t make a follow up. Because some can forget and some are hard-headed people many are there they don’t know that taking cure of a child is something important.
