# Supplementary material for: Community acceptability of Seasonal Malaria Chemoprevention of morbidity and mortality in young children: A qualitative study in the Upper West Region of Ghana
Source: PLoS One. 2019 May 17;14(5):e0216486. doi: 10.1371/journal.pone.0216486 (PMC6524792; doi:10.1371/journal.pone.0216486)
Supplement: S1 File — (ZIP) [file pone.0216486.s001.zip › Study data set-Nvivo coding/Perceptions of other family members.docx]

**Perceptions of other family members**

**IDIs with mothers**

[<Internals\\IDIs health workers\\IDIs mothers\\IDI 18 year old mother-Tanziir>](file:///C:\Users\chatio\Desktop\Save%20in%20drive\studies\PK\SMC%20report\Final%20SMC%20report\Mothers\Acceptability\d9286b71-0d0b-4b69-a3d3-30b1fce77d91) - § 1 reference coded [3.33% Coverage]

Reference 1 - 3.33% Coverage

Q: But those staying with you, your yard people, what did they say concerning this drug?

R: Anytime I collect the drug, they normally say I should do well and give the drug to the child.

Q: Why do they say that?

R: They themselves have seen that the child used to get sick always but when I started giving him the drug, it is not like that again. That is why they normally press me to give him.

[<Internals\\IDIs health workers\\IDIs mothers\\IDI 20 year old mother-Gbier>](file:///C:\Users\chatio\Desktop\Save%20in%20drive\studies\PK\SMC%20report\Final%20SMC%20report\Mothers\Acceptability\6d8a376a-063d-4b88-94d3-30b1fe15cc3a) - § 1 reference coded [0.99% Coverage]

Reference 1 - 0.99% Coverage

Q: Does your husband like these drugs?

R: Yes

Q: Does your husband want his child to be receiving the drugs?

R: Yes

[<Internals\\IDIs health workers\\IDIs mothers\\IDI 20 yearold mother-Berwong1>](file:///C:\Users\chatio\Desktop\Save%20in%20drive\studies\PK\SMC%20report\Final%20SMC%20report\Mothers\Acceptability\c8dc56f9-1da6-4c14-88d3-30b1fe3e4370) - § 1 reference coded [2.21% Coverage]

Reference 1 - 2.21% Coverage

Q: Can you tell me what your husband or any relative think or say about this medicine?

R: They are happy about the medicine because they have never seen my child been sick again since the child took the drug.

[<Internals\\IDIs health workers\\IDIs mothers\\IDI 26 year old mother-Newtown>](file:///C:\Users\chatio\Desktop\Save%20in%20drive\studies\PK\SMC%20report\Final%20SMC%20report\Mothers\Acceptability\d2cb5fdf-6b7b-4d12-b6d3-30b1fe4a2a75) - § 2 references coded [5.79% Coverage]

Reference 1 - 2.06% Coverage

R. There have not said anything but their attitude initially showed that they really wanted the drugs

[<Internals\\IDIs health workers\\IDIs mothers\\IDI 26 yearold mother-Eremon Tangzu (Autosaved)>](file:///C:\Users\chatio\Desktop\Save%20in%20drive\studies\PK\SMC%20report\Final%20SMC%20report\Mothers\Acceptability\dfa639bf-3760-46df-96d3-30b1fe5ad475) - § 1 reference coded [3.18% Coverage]

Reference 1 - 3.18% Coverage

Q: What did your husband say concerning the drug they gave to your child?

R: He didn’t say anything.

Q: Is he aware that the child is taken the drug and he didn’t say anything?

R: Yes.

Q: But does he want his child to be taking the drug?

R: Yes.

[<Internals\\IDIs health workers\\IDIs mothers\\IDI 27 year old mother-Newtown>](file:///C:\Users\chatio\Desktop\Save%20in%20drive\studies\PK\SMC%20report\Final%20SMC%20report\Mothers\Acceptability\6ada4bad-92df-4de8-93d3-30b1fe7503c8) - § 1 reference coded [3.55% Coverage]

Reference 1 - 3.55% Coverage

**Q.** What does your husband say about the drugs?

R. He supports the intervention, whenever they brings it, he is always eager for me to give them to the child.

[<Internals\\IDIs health workers\\IDIs mothers\\IDI 28 year old mother-Berwong>](file:///C:\Users\chatio\Desktop\Save%20in%20drive\studies\PK\SMC%20report\Final%20SMC%20report\Mothers\Acceptability\74cb6986-f6d6-4621-99d3-30b1fe80eacc) - § 4 references coded [2.83% Coverage]

Reference 1 - 0.66% Coverage

Q: What did your husband say concerning this drug?

R: He didn’t say anything.

Reference 2 - 1.20% Coverage

Q: What did your relatives say concerning this drug?

R: For that one they are happy one of sisters asked me one day how he is when I told her that a certain drug came and took it he not getting sick she said that drugs are powerful than each other.

Reference 3 - 0.23% Coverage

[<Internals\\IDIs health workers\\IDIs mothers\\IDI 30 year old mother-BagriE>](file:///C:\Users\chatio\Desktop\Save%20in%20drive\studies\PK\SMC%20report\Final%20SMC%20report\Mothers\Acceptability\f4681a8b-1b78-4cab-add3-30b1fe8f3281) - § 1 reference coded [2.67% Coverage]

Reference 1 - 2.67% Coverage

R. As for my husband, he usually ask me whether i have given the child the drug? If i say no, then he gets angry at me for me to give the child the drug. So he knows that the drug is very helpful to our child**.**

Q. Meaning your husband supported the administration of this drug?

R. Yes, he knew it was very helpful to our child

[<Internals\\IDIs health workers\\IDIs mothers\\IDI 30 year old mother-Kolbugnuor>](file:///C:\Users\chatio\Desktop\Save%20in%20drive\studies\PK\SMC%20report\Final%20SMC%20report\Mothers\Acceptability\452bf184-0cec-4af6-95d3-30b1feee696f) - § 1 reference coded [3.11% Coverage]

Reference 1 - 3.11% Coverage

Q. What do people especially your husband say about these drugs?

R. When i told him about the drug, he told me to try and let the the child take all the drugs as expected.

[<Internals\\IDIs health workers\\IDIs mothers\\IDI 30 year old mother-Newtown>](file:///C:\Users\chatio\Desktop\Save%20in%20drive\studies\PK\SMC%20report\Final%20SMC%20report\Mothers\Acceptability\1950e505-2542-4323-b9d3-30b1fef7ef36) - § 1 reference coded [3.27% Coverage]

Reference 1 - 3.27% Coverage

Q: What does your husband say about this medicine they gave you?

R: He is happy that the child is taking it.

Q: What are the views of people you stay with about this medicine?

R: They don`t say anything.

[<Internals\\IDIs health workers\\IDIs mothers\\IDI 30 year old mother-Tuma>](file:///C:\Users\chatio\Desktop\Save%20in%20drive\studies\PK\SMC%20report\Final%20SMC%20report\Mothers\Acceptability\713306ab-8ca2-47cb-96d3-30b1feff1321) - § 1 reference coded [4.20% Coverage]

Reference 1 - 4.20% Coverage

Q: Those staying with you and your husband, what did they say concerning this drug?

R: Ok, my husband never said anything concerning this drug, when the child took and fell ill he came and if the following day if he is still ill we should take him to hospital, but he didn’t say anything concerning the drug.

Q: But, do you think your husband likes this medicine?

R: Ok, he likes it since it helps us from going to hospital all the times.

[<Internals\\IDIs health workers\\IDIs mothers\\IDI 31 year old mother-Eremon Tangzu>](file:///C:\Users\chatio\Desktop\Save%20in%20drive\studies\PK\SMC%20report\Final%20SMC%20report\Mothers\Acceptability\07b07256-641a-4fb8-9fd3-30b1ff147f2e) - § 2 references coded [3.82% Coverage]

Reference 1 - 2.18% Coverage

Q: What does your husband say about this drug?

R: Ok, I can say my husband is someone who travels so he is not always with us so he won’t know all these things.

Q: But did you tell him about the drug?

R: When he did not hear about the child’s sickness that he asked and I told him that they brought some drug to give to the children to protect them from the malaria which he took.

Q: So when you told him what did he say?

R: He said the people who gave the drug did well.

Reference 2 - 1.64% Coverage

Q: What about those you are staying with?

R: That is what I just finished talking about. We used to wake up in the night and ran to the hospital because your colleague’s child is sick but when they started giving the drug we don’t knock on nurses doors again in the night.

Q: So the people you staying with do they like this drug?

R: Yes, they want it.

[<Internals\\IDIs health workers\\IDIs mothers\\IDI 31 year old mother-Kolbugnuor>](file:///C:\Users\chatio\Desktop\Save%20in%20drive\studies\PK\SMC%20report\Final%20SMC%20report\Mothers\Acceptability\e249c295-786b-44e2-96d3-30b1ff33714a) - § 2 references coded [4.59% Coverage]

Reference 1 - 2.71% Coverage

Q; Can you tell me the views of your husband towards this medicine?

R; He don`t know anything about this medicine.

Q; Haven`t you told him anything about this medicine?

R; He is not aware the child has taken any medicine.

Reference 2 - 1.89% Coverage

Q; Are your yard people aware of this medicine?

R; Yes.

Q; What do they say about the medicine?

R; They say the medicine is very good for the children.

[<Internals\\IDIs health workers\\IDIs mothers\\IDI 31 year old mother-Tuma>](file:///C:\Users\chatio\Desktop\Save%20in%20drive\studies\PK\SMC%20report\Final%20SMC%20report\Mothers\Acceptability\70adc82b-bbde-47e1-8fd3-31c514093d39) - § 1 reference coded [3.46% Coverage]

Reference 1 - 3.46% Coverage

Q: But those staying with you, your yard people, what did they say concerning this medicine?

R: Ok, they are saying that the way my child was and they started giving him the medicine, that he looks nice, that the medicine is good.

[<Internals\\IDIs health workers\\IDIs mothers\\IDI 32 year old mother-Gbier>](file:///C:\Users\chatio\Desktop\Save%20in%20drive\studies\PK\SMC%20report\Final%20SMC%20report\Mothers\Acceptability\169607a2-22bc-48ff-b4d3-31cc71f08747) - § 1 reference coded [3.12% Coverage]

Reference 1 - 3.12% Coverage

Q: what are your family members or friends saying about this malaria drugs, especially your husband?

R: as for my husband he had interest in the drugs and so even if I am not there he normally gives the drugs to the child.

Q: Does he like the drugs?

R: yes, because he himself sometimes gives drugs the drugs to the child.

[<Internals\\IDIs health workers\\IDIs mothers\\IDI 35 year old mother-Bagri>](file:///C:\Users\chatio\Desktop\Save%20in%20drive\studies\PK\SMC%20report\Final%20SMC%20report\Mothers\Acceptability\4d2676eb-d04a-4099-a3d3-30b1ff7f9d5c) - § 2 references coded [4.14% Coverage]

Reference 1 - 2.89% Coverage

R: Some time I was not there it was a market day and Saani came here he himself took the child to the old house and met him for the medicine, and kept the rest when I returned he narrated to me how to administer it and I said I have been doing it so I know it.

Q: So, is your husband like this drugs or not?

R: He likes it, if he doesn’t he won’t look for it and kept it for me.

[<Internals\\IDIs health workers\\IDIs mothers\\IDI 35 year old mother-Tanziir>](file:///C:\Users\chatio\Desktop\Save%20in%20drive\studies\PK\SMC%20report\Final%20SMC%20report\Mothers\Acceptability\f4a9dcf4-f49d-44b0-9bd3-30b1ff9e8f9d) - § 2 references coded [2.70% Coverage]

Reference 1 - 1.10% Coverage

Q: Your husband what does he says concerning this medicine?

R: If they bring the drug he put me on pressure to give him.

Reference 2 - 1.59% Coverage

Q: Why does he always in a hurry you should give the child the drug?

R: If the child is sick all of us will be troubled that is why he is always in a hurry I should give him.

[<Internals\\IDIs health workers\\IDIs mothers\\IDI 36 year old mother-Bagri>](file:///C:\Users\chatio\Desktop\Save%20in%20drive\studies\PK\SMC%20report\Final%20SMC%20report\Mothers\Acceptability\8687b6b6-f1c5-444b-acd3-30b1ffbfe296) - § 1 reference coded [0.60% Coverage]

Reference 1 - 0.60% Coverage

Q: Like your husband what did he say about the drugs?

R: He did not say anything.

[<Internals\\IDIs health workers\\IDIs mothers\\IDI 37 year old mother-Kolbugnuor>](file:///C:\Users\chatio\Desktop\Save%20in%20drive\studies\PK\SMC%20report\Final%20SMC%20report\Mothers\Acceptability\e04a7c1d-5fa7-4576-8dd3-30b1ffd54e38) - § 2 references coded [2.17% Coverage]

Reference 1 - 1.00% Coverage

R; He is happy the children are taking the medicine.

Reference 2 - 1.17% Coverage

R; They are also taking this medicine and are happy about it.

[<Internals\\IDIs health workers\\IDIs mothers\\IDI 50 year old mother-Ngman-gbil>](file:///C:\Users\chatio\Desktop\Save%20in%20drive\studies\PK\SMC%20report\Final%20SMC%20report\Mothers\Acceptability\2c4c0db0-dba2-4de3-bcd3-30b1ffdc73e0) - § 4 references coded [3.09% Coverage]

Reference 1 - 0.34% Coverage

R: Ok, they said the medicine is good.
