# Supplementary material for: Community acceptability of Seasonal Malaria Chemoprevention of morbidity and mortality in young children: A qualitative study in the Upper West Region of Ghana
Source: PLoS One. 2019 May 17;14(5):e0216486. doi: 10.1371/journal.pone.0216486 (PMC6524792; doi:10.1371/journal.pone.0216486)
Supplement: S1 File — (ZIP) [file pone.0216486.s001.zip › Study data set-Nvivo coding/Prevalence of malaria among under five children.docx]

**Views of stakeholders on prevalence of malaria among under five children**

**IDIs with mothers**

[<Internals\\IDIs health workers\\IDIs mothers\\IDI 18 year old mother-Tanziir>](file:///C:\Users\chatio\Desktop\Save%20in%20drive\studies\PK\SMC%20report\Final%20SMC%20report\Mothers\Disease%20and%20prevalence\d9286b71-0d0b-4b69-a3d3-30b1fce77d91) - § 1 reference coded [6.96% Coverage]

Reference 1 - 6.96% Coverage

Q: You mention malaria, take malaria this year and the past years, is it they still the same or there is a difference?

R: In our community, there is a difference.

Q: What is the difference is it decreasing or increasing?

R: It is decreasing.

Q: What is causing the decreased?

R: It is decreasing because of the drug they are giving to us and our rooms they came to spray. These are making malaria cases to reduce here. (IDI-18 year old mother)

Q: If you take malaria cases among children under this year and the past years are they still the same or there is difference?

R: There is difference.

Q: The difference, a decreased or an increased?

R: It has reduced.

[<Internals\\IDIs health workers\\IDIs mothers\\IDI 20 year old mother-Gbier>](file:///C:\Users\chatio\Desktop\Save%20in%20drive\studies\PK\SMC%20report\Final%20SMC%20report\Mothers\Disease%20and%20prevalence\6d8a376a-063d-4b88-94d3-30b1fe15cc3a) - § 1 reference coded [3.52% Coverage]

Reference 1 - 3.52% Coverage

Q; Comparing the previous years and the present, will you say there is improvement in preventing or reducing the effect of malaria in children?

R: Yes. There is improvement because of the drugs they came and distributed to us.

Q: Do you have something you do that is causing the reduction of malaria infection?

R: No. There is nothing else that we do but just because of the mosquito net we sleep in and the malaria drug.

[<Internals\\IDIs health workers\\IDIs mothers\\IDI 20 yearold mother-Berwong1>](file:///C:\Users\chatio\Desktop\Save%20in%20drive\studies\PK\SMC%20report\Final%20SMC%20report\Mothers\Disease%20and%20prevalence\c8dc56f9-1da6-4c14-88d3-30b1fe3e4370) - § 1 reference coded [12.32% Coverage]

Reference 1 - 12.32% Coverage

Q: Considering malaria, for some years past and now has there been any changes or it is still the same?

R: There has been an improvement; we no longer experience it as before.

Q: What brought about the improvement?

R: This improvement has come because of the malaria drug (SP&AQ) they gave to us to give to our children.

Q: Apart from the drug given to the children, what other thing has brought about the reduction of malaria in the community?

R: At first the children experience fever but when they started giving the drug, they don`t suffer from malaria again.

Q: What are the things you do which brought about the reduction in malaria?

R: We now sleep under mosquito nets and cover our water.

Q: With children that are not up to five years in your community, has there been improvement in malaria infection or not?

R: We no longer experience high cases as before.

[<Internals\\IDIs health workers\\IDIs mothers\\IDI 26 year old mother-Newtown>](file:///C:\Users\chatio\Desktop\Save%20in%20drive\studies\PK\SMC%20report\Final%20SMC%20report\Mothers\Disease%20and%20prevalence\d2cb5fdf-6b7b-4d12-b6d3-30b1fe4a2a75) - § 2 references coded [5.61% Coverage]

Reference 1 - 0.20% Coverage

R. there is a decline

Reference 2 - 5.42% Coverage

Q. What do you do in this your community that contributes to the decline of malaria cases?

R. Cleaning of the surroundings and the SMC drugs

Q. For the past two years, has the cases of malaria on a rise or decrease with respect to children under five?

R. There is a decrease

[<Internals\\IDIs health workers\\IDIs mothers\\IDI 26 yearold mother-Eremon Tangzu (Autosaved)>](file:///C:\Users\chatio\Desktop\Save%20in%20drive\studies\PK\SMC%20report\Final%20SMC%20report\Mothers\Disease%20and%20prevalence\dfa639bf-3760-46df-96d3-30b1fe5ad475) - § 1 reference coded [10.78% Coverage]

Reference 1 - 10.78% Coverage

Q: If you look at the malaria disease of the past years compare to now is there a change or it is still the same?

R: A change is there.

Q: Is it reducing or increasing?

R: It is reducing.

Q: What are you doing in this community to cause the reduction?

R: It is this drug (SP&AQ) they brought initially when you are passing by the hospital you will see many mothers but now if go there mothers are not many again it is rather the elderly once to mean the elderly the malaria is worrying us.

Q: The adults and the children that did not take the drug, if you look at the past years and now do you think it is decreasing or it is still like that.

R: Those who have not taken the drug it is increasing.

Q: Why is it increasing?

R: The virus is always in the body growing so it is increasing among them.

[<Internals\\IDIs health workers\\IDIs mothers\\IDI 27 year old mother-Newtown>](file:///C:\Users\chatio\Desktop\Save%20in%20drive\studies\PK\SMC%20report\Final%20SMC%20report\Mothers\Disease%20and%20prevalence\6ada4bad-92df-4de8-93d3-30b1fe7503c8) - § 1 reference coded [2.71% Coverage]

Reference 1 - 2.71% Coverage

Q. Is the problem stills the same as in the past?

R. There has been a decrease especially on the children who took the drugs.

Q. What of the children who did not take the drugs?

R. There have been a bit of reduction but among we the adults, I cannot tell.

[<Internals\\IDIs health workers\\IDIs mothers\\IDI 28 year old mother-Berwong>](file:///C:\Users\chatio\Desktop\Save%20in%20drive\studies\PK\SMC%20report\Final%20SMC%20report\Mothers\Disease%20and%20prevalence\74cb6986-f6d6-4621-99d3-30b1fe80eacc) - § 2 references coded [8.26% Coverage]

Reference 1 - 3.20% Coverage

Q: So if you look at the malaria disease in this community, the past years and now is there a change or it is still the same?

R: It has change.

Q: The change is it decreasing or increasing?

R: It is decreasing.

Q: What is causing the reduction?

R: I can say this drug that came to protect us.

[<Internals\\IDIs health workers\\IDIs mothers\\IDI 30 year old mother-BagriE>](file:///C:\Users\chatio\Desktop\Save%20in%20drive\studies\PK\SMC%20report\Final%20SMC%20report\Mothers\Disease%20and%20prevalence\f4681a8b-1b78-4cab-add3-30b1fe8f3281) - § 3 references coded [14.27% Coverage]

Reference 1 - 4.68% Coverage

R. We can say that with the drugs that were given to children this year even though were the elderly ones where not part, we have gottten some relief on the side of the children. I can confidently say that for this year, my child has not vomited or having a very high body temperature not until recent he had i little high body temperature but was treated when i took him to the CHPS compound. But i can say there is a reduction in the cases we use to have as compared to this year.

Reference 2 - 4.14% Coverage

Q. If you should take into consideration everybody in this community regardless of been a child or an adult, can you say that the cases of malaria has declined or increased?

R. As for the children it has reduced because of the drugs they took, but we the elderly are making good use of the mosquito nets that were distributed to us. Generally, i can say there has been a decline in the cases of malaria in this community.

Reference 3 - 5.45% Coverage

Q. You said there have been a decline because of mosquito nets you use, in your mind what do you think is the contributory factor to this reduction of malaria cases i your community?

R. It also depends on how well you take care of yourself. When you watch carefully what you eat and the water that you drink. Most importantly, you have to keep your water containers clean this i believe has contributed to the reduction of malaria cases.

[<Internals\\IDIs health workers\\IDIs mothers\\IDI 30 year old mother-Eremon Tangzu>](file:///C:\Users\chatio\Desktop\Save%20in%20drive\studies\PK\SMC%20report\Final%20SMC%20report\Mothers\Disease%20and%20prevalence\99dc5250-fb67-4d4d-8ad3-30b1fea49eb2) - § 1 reference coded [4.48% Coverage]

Reference 1 - 4.48% Coverage

R: Changes are there.

Q: Is it increasing or decreasing.

R: It is decreasing.

Q: What shows that it is decreasing?

R: The way our children were dying they are no more dying that way.

[<Internals\\IDIs health workers\\IDIs mothers\\IDI 30 year old mother-Gbier>](file:///C:\Users\chatio\Desktop\Save%20in%20drive\studies\PK\SMC%20report\Final%20SMC%20report\Mothers\Disease%20and%20prevalence\90c36595-6809-4c06-96d3-30b1fee4e4c2) - § 1 reference coded [15.28% Coverage]

Reference 1 - 15.28% Coverage

Q; Comparing the previous years and the present, will you say there is improvement in preventing or reducing the effect of malaria?

R: Yes, it has reduced.

Q: What measures have you put in place to chop this success?

R: We started clearing nearby bushes, desilting stagnant waters and sleeping under mosquito nets.

Q: If you look among children under five, will you say there is reduction of malaria in their systems?

R: Yes, there is reduction because of the malaria drugs they distributed to our children.

Q: What did it do to your children to reduce malaria cases?

R: The drugs cured all malaria cases in our children.

Q: In what way?

R: Our children no longer fall sick. These drugs gave good health and make the child intelligent.

Q: Why do you say that the drugs give good health to your child?

R: If you look at the previous health of the child and the present stature, it means this current malaria drugs boost healthy growth to the child as compare to other drugs. The drugs now remove all malaria diseases that used to slow down the child’s activeness.

[<Internals\\IDIs health workers\\IDIs mothers\\IDI 30 year old mother-Kolbugnuor>](file:///C:\Users\chatio\Desktop\Save%20in%20drive\studies\PK\SMC%20report\Final%20SMC%20report\Mothers\Disease%20and%20prevalence\452bf184-0cec-4af6-95d3-30b1feee696f) - § 3 references coded [7.74% Coverage]

Reference 1 - 1.65% Coverage

Q. Over the years, have there been any improvement?

R. Since they started giving these drugs, those who took the drugs has not often complain of malaria

Reference 2 - 4.44% Coverage

Q. Aside what you mentioned, are there any other factors/

R. It is because we also sleep under treated mosquito bed nets

[<Internals\\IDIs health workers\\IDIs mothers\\IDI 30 year old mother-Newtown>](file:///C:\Users\chatio\Desktop\Save%20in%20drive\studies\PK\SMC%20report\Final%20SMC%20report\Mothers\Disease%20and%20prevalence\1950e505-2542-4323-b9d3-30b1fef7ef36) - § 2 references coded [4.33% Coverage]

Reference 1 - 1.62% Coverage

Q: What is the community doing to protect themselves against malaria?

R: We sleep under mosquito nets.

Reference 2 - 2.71% Coverage

Q: Have the situation change/remain the same currently among children under five?

R: It has changed; there has been a reduction since the children started taken the drugs.

[<Internals\\IDIs health workers\\IDIs mothers\\IDI 30 year old mother-Tuma>](file:///C:\Users\chatio\Desktop\Save%20in%20drive\studies\PK\SMC%20report\Final%20SMC%20report\Mothers\Disease%20and%20prevalence\713306ab-8ca2-47cb-96d3-30b1feff1321) - § 1 reference coded [7.39% Coverage]

Reference 1 - 7.39% Coverage

Q: You mention malaria disease, take malaria this year and the past years are they still the same or there are changes?

R: For this year it is about 3 months, for children there are changes. The children that they were going round giving them some drugs who used to go to hospital almost every now it is somehow better.

[<Internals\\IDIs health workers\\IDIs mothers\\IDI 31 year old mother-Eremon Tangzu>](file:///C:\Users\chatio\Desktop\Save%20in%20drive\studies\PK\SMC%20report\Final%20SMC%20report\Mothers\Disease%20and%20prevalence\07b07256-641a-4fb8-9fd3-30b1ff147f2e) - § 4 references coded [11.91% Coverage]

Reference 1 - 2.60% Coverage

Q: The malaria disease you mention if check now is it change or it is still like the past years?

R: I can say there is a change among the children but we us adults it is still the same.

Q: Let take the past two years and now is it reduced or still the same?

R: That is what I said, it has reduced but I have realized a decrease among children but the in adults it is still the same.

Q: What caused the reduction in children?

R: The children you have done well by giving them the drug. So far, it has decreased in children but we the grownups it is still the same.

[<Internals\\IDIs health workers\\IDIs mothers\\IDI 31 year old mother-Tuma>](file:///C:\\Users\\chatio\\Desktop\\Save%20in%20drive\\studies\\PK\\SMC%20report\\Final%20SMC%20report\\Mothers\\Disease%20and%20prevalence\\70adc82b-bbde-47e1-8fd3-31c514093d39) - § 2 references coded [9.40% Coverage]

Reference 1 - 4.81% Coverage

R: It used to worry people at first but when they shared the nets (mosquito nets) it is no more doing that.

Q: It is because of the nets alone that stopped the malaria or there is something that you do to?

R: Also, it got to a time they brought some medicine to the children, to me my child’s body was just reducing and I took him to the hospital and they said nothing is worrying him but gave me some food to be preparing for him for about one week it was still like that, but that medicine they brought I won’t tell a lie against that medicine when the child took the medicine he is healthy till now

Q: But the malaria among children looking at the past two years compare to this year is it reducing, or increasing or it is still the same?

R: Reduction is there; it was affecting my children frequently and I go to hospital, now I normally go for health insurance for a full year and I don’t go to hospital. Unless some disease that is there, it makes the child’s body itches and you see some boils, we call it ‘’nyaaro-ur’’ they normally appear, but the local medicine that they show as that you always add salt to water and rob the person, when I do that it normally go.

[<Internals\\IDIs health workers\\IDIs mothers\\IDI 32 year old mother-Gbier>](file:///C:\Users\chatio\Desktop\Save%20in%20drive\studies\PK\SMC%20report\Final%20SMC%20report\Mothers\Disease%20and%20prevalence\169607a2-22bc-48ff-b4d3-31cc71f08747) - § 2 references coded [10.16% Coverage]

Reference 1 - 5.47% Coverage

R: it has decrease drastically.

It is not like those days in which children are admitted at the hospital anyhow.

If a child use to fall sick and you give him/her this particular malaria drugs he or she will recover quickly.

Q: a part from the malaria drugs given to the children and sleeping in the mosquito net, is there any methods that contribute to the reduction of the malaria infection?

R: well, they used to come and spray our homes with some chemicals (insecticides)

[<Internals\\IDIs health workers\\IDIs mothers\\IDI 34 year old mother-Tuma>](file:///C:\Users\chatio\Desktop\Save%20in%20drive\studies\PK\SMC%20report\Final%20SMC%20report\Mothers\Disease%20and%20prevalence\49e33dc3-05d2-443e-b6d3-30b1ff71558e) - § 3 references coded [11.50% Coverage]

Reference 1 - 2.05% Coverage

R: Changes are there.

Q: What are the changes?

R: Changes that are there are: they brought some medicine for us. The medicine that they brought to as like if they bring it in the morning the following day morning we have to give it to the child at the same time. They gave as white and yellow

Q: Considering children under five years, now is the disease still the same comparing it with the past year or years?

R: No, now it has reduced because some were not practicing what we were told to do, they thought they were punishing people but now we have change.

[<Internals\\IDIs health workers\\IDIs mothers\\IDI 35 year old mother-Tanziir>](file:///C:\Users\chatio\Desktop\Save%20in%20drive\studies\PK\SMC%20report\Final%20SMC%20report\Mothers\Disease%20and%20prevalence\f4a9dcf4-f49d-44b0-9bd3-30b1ff9e8f9d) - § 2 references coded [2.20% Coverage]

Reference 1 - 0.97% Coverage

R: It is changing.

Q: It is changing, does it means it is increasing or decreasing?

R: It is decreasing.

Reference 2 - 1.23% Coverage

R: The medicine that is out and they are shearing is the reason.

Q: Are elderly people also collecting?

R: No, they give to children.

[<Internals\\IDIs health workers\\IDIs mothers\\IDI 36 year old mother-Bagri>](file:///C:\Users\chatio\Desktop\Save%20in%20drive\studies\PK\SMC%20report\Final%20SMC%20report\Mothers\Disease%20and%20prevalence\8687b6b6-f1c5-444b-acd3-30b1ffbfe296) - § 2 references coded [2.35% Coverage]

Reference 1 - 0.58% Coverage

R: Yes, for this year it has reduced, it does not worry the children that much.

Reference 2 - 1.77% Coverage

Q: What shows that, the past year it was worrying you?

R: Because, the past year we were not getting the medicine, but this year we have the mosquito nets, they are spraying our rooms and giving as the medicine, so this year it has reduced.

[<Internals\\IDIs health workers\\IDIs mothers\\IDI-23 year old mother-Berwong>](file:///C:\Users\chatio\Desktop\Save%20in%20drive\studies\PK\SMC%20report\Final%20SMC%20report\Mothers\Disease%20and%20prevalence\1361ca8d-5b5d-4820-88d3-3eaa2e45ad3d) - § 3 references coded [2.47% Coverage]

Reference 1 - 0.52% Coverage

R: ok if you look the past and now, there have been some improvement. Now it is not worrying us like before.

Reference 2 - 1.55% Coverage

Q: why do you say it has improved now, or what have you been doing to cause the improvement?

R: now there have been health education on how to keep our surrounding clean, construction of toilets and also, we have been told to sleep under mosquito net and because of these things the occurrence of the disease has minimized.

**FGDs with fathers and mothers**

[<Internals\\FGDs\\FGD fathers with children under five-Tanziir>](file:///C:\Users\chatio\Desktop\Save%20in%20drive\studies\PK\SMC%20report\Final%20SMC%20report\FGDs\Diseases%20and%20prevelance\12582ff3-0c50-46c7-82d3-3403c9ad39ce) - § 4 references coded [2.06% Coverage]

Reference 1 - 0.41% Coverage

R: Malaria has reduced and it is because of the drug they gave to the children and our rooms they normally come and spray.

Reference 2 - 0.25% Coverage

No.6

R: It has reduced; it is a few children still suffer.

Reference 3 - 0.36% Coverage

R: Malaria infection among children under five to me I have seen a drastic reduction.

Reference 4 - 1.04% Coverage

Q: Then can you tell me why the reduction among the under five children?

R: I can say the mosquito nets, the pray (AgAMAL control programme) and the drug (SP &AQ) given to the children caused the reduction of malaria among the under five children.

[<Internals\\FGDs\\FGD fathers with children under five-Zambo>](file:///C:\Users\chatio\Desktop\Save%20in%20drive\studies\PK\SMC%20report\Final%20SMC%20report\FGDs\Diseases%20and%20prevelance\903102be-0a51-4b0e-93d3-3403caff3dbb) - § 1 reference coded [0.60% Coverage]

Reference 1 - 0.60% Coverage

R; They come to spray our rooms and we also sleep under mosquito net.

[<Internals\\FGDs\\FGD mothers with children under five-Tanziir>](file:///C:\Users\chatio\Desktop\Save%20in%20drive\studies\PK\SMC%20report\Final%20SMC%20report\FGDs\Diseases%20and%20prevelance\7933f35b-9705-4934-95d3-3403cb0fe604) - § 3 references coded [2.14% Coverage]

Reference 1 - 0.32% Coverage

No.1

R: They also brought some chemicals and spray our rooms so the mosquitoes are few.

Reference 2 - 1.26% Coverage

R: It has reduced.

Q: Why the reduction?

R: It is because of the drug the volunteer brought for the four months contributed to the reduction.

No.6

R: The malaria disease now they have started giving us drugs we are no more experiencing the bad things, children used to get sick always and we rush them to hospital, now that is not happening again.

Reference 3 - 0.56% Coverage

R: It is true since they brought this drug we are not experiencing children getting sick like that.

Q: So is it reducing or increasing?

R: it is reducing.

[<Internals\\FGDs\\FGD-fathers with children under five-Gbier>](file:///C:\Users\chatio\Desktop\Save%20in%20drive\studies\PK\SMC%20report\Final%20SMC%20report\FGDs\Diseases%20and%20prevelance\72a70c42-22e7-42d9-a8d3-3ea9fb2b287c) - § 2 references coded [1.76% Coverage]

Reference 1 - 1.10% Coverage

No.6

R: The malaria disease you are talking about is the result of dirty waters and rubbish, we used to carry our children to hospital always just because we were not taught. But now we are aware of it, so malaria is reducing.

Reference 2 - 0.67% Coverage

No.7

R: Our bathroom waters we cover them and make our places clean so that mosquitoes will not worry us again, what I have seen is that.

[<Internals\\FGDs\\FGD-mothers with children under five-Gbier>](file:///C:\Users\chatio\Desktop\Save%20in%20drive\studies\PK\SMC%20report\Final%20SMC%20report\FGDs\Diseases%20and%20prevelance\18f67d6c-f146-473c-83d3-3ea9fb47b8e4) - § 1 reference coded [1.32% Coverage]

Reference 1 - 1.32% Coverage

R; I send the child to the hospital for treatment.

NUMBER FOUR

R; At first I use herbs but now I send my child to the hospital when he is sick of malaria.

**IDIs with health volunteers**

[<Internals\\IDIs health volunteers\\IDI 25 year old health volunteer-Eremon Tangzu>](file:///C:\Users\chatio\Desktop\Save%20in%20drive\studies\PK\SMC%20report\Final%20SMC%20report\Volunteers\Diseases%20and%20prevelance\e21b7ca5-f80d-4acc-8fd3-2cbcfe7276ff) - § 1 reference coded [1.16% Coverage]

Reference 1 - 1.16% Coverage

R: It has reduced.

Q: What cause the reduction among children under five?

R: It is the cause of the mosquito nets.

Q: Presently is it only the mosquito nets that are causing the reduction in the cases of malaria among the children under five?

R: The mosquito nets and the SMC drugs.

[<Internals\\IDIs health volunteers\\IDI 34 year old Health volunteer-Ngman-gbil>](file:///C:\Users\chatio\Desktop\Save%20in%20drive\studies\PK\SMC%20report\Final%20SMC%20report\Volunteers\Diseases%20and%20prevelance\9792d99e-569d-43a4-aad3-3404088aea56) - § 2 references coded [2.09% Coverage]

Reference 1 - 0.50% Coverage

R: In my mind last year malaria was worrying children badly but this year because of the drug they gave to them we are not seeing malaria with them again.

Reference 2 - 1.58% Coverage

R: They last gave as some CBA medicine if the child is sick we normally give it to the child if it is not better we then take the child to hospital. They also gave some medicine, amodiaquine.

Q: Aside CBA and hospital is there something you do when a child is sick of malaria?

R: Some people go round and ask of children who are not up to 5years, I know if they are sick they normally take them to hospital.

Q: Who are those people?

R: I don’t know them anyway but they go round.

[<Internals\\IDIs health volunteers\\IDI 35 year old Health volunteer-Bagri>](file:///C:\Users\chatio\Desktop\Save%20in%20drive\studies\PK\SMC%20report\Final%20SMC%20report\Volunteers\Diseases%20and%20prevelance\570786a5-e7ba-4707-a3d3-340408a2b74c) - § 1 reference coded [0.93% Coverage]

Reference 1 - 0.93% Coverage

R: The malaria disease among children under five years has reduced. Those who are rather above the five years they are rather the most affected, here if you take them to hospital anytime it is always malaria.

[<Internals\\IDIs health volunteers\\IDI 35 year old Health volunteer-Newtown>](file:///C:\Users\chatio\Desktop\Save%20in%20drive\studies\PK\SMC%20report\Final%20SMC%20report\Volunteers\Diseases%20and%20prevelance\e62ddbb9-5b7b-4af3-aed3-340408b8247e) - § 1 reference coded [1.11% Coverage]

Reference 1 - 1.11% Coverage

R. There has been a reduction

Q. What do people do when their children under five years get affected by malaria?

R. They usually take the child to the hospital

[<Internals\\IDIs health volunteers\\IDI 35 year old Health volunteer-Tuma>](file:///C:\Users\chatio\Desktop\Save%20in%20drive\studies\PK\SMC%20report\Final%20SMC%20report\Volunteers\Diseases%20and%20prevelance\ec8b1f3e-8b41-4285-89d3-340408c8cd1c) - § 1 reference coded [1.12% Coverage]

Reference 1 - 1.12% Coverage

Q: Why do you think it is still the same?

R: When they give us the drug and it finishes we have to always go back, but when we go to hospital and they give to us when we come and take it and feel better we normally the disease is gone, but when you are sick and go back they will tell you that it is malaria again.

[<Internals\\IDIs health volunteers\\IDI 36 year old Health volunteer-Tanziir>](file:///C:\Users\chatio\Desktop\Save%20in%20drive\studies\PK\SMC%20report\Final%20SMC%20report\Volunteers\Diseases%20and%20prevelance\1a01ef21-0554-43f3-acd3-340408de3ab4) - § 1 reference coded [0.68% Coverage]

Reference 1 - 0.68% Coverage

R: for the children, the past years they were getting sick regularly but this year especially from the farming season that was the time they brought the drug to help us.

[<Internals\\IDIs health volunteers\\IDI 45 year old Health volunteer- Kolbugnuor>](file:///C:\Users\chatio\Desktop\Save%20in%20drive\studies\PK\SMC%20report\Final%20SMC%20report\Volunteers\Diseases%20and%20prevelance\db73d9f0-71e5-4376-86d3-340409449585) - § 1 reference coded [0.15% Coverage]

Reference 1 - 0.15% Coverage

R. There has been a reduction and it is because of the SMC drug that the children have received in the communities.

[<Internals\\IDIs health volunteers\\IDI 45 year old Health volunteer-Gbier>](file:///C:\Users\chatio\Desktop\Save%20in%20drive\studies\PK\SMC%20report\Final%20SMC%20report\Volunteers\Diseases%20and%20prevelance\dfa339b5-1827-441d-9bd3-340409553f66) - § 1 reference coded [0.74% Coverage]

Reference 1 - 0.74% Coverage

R. On the side of the children, it is because of the SMC drugs that they took and also the spraying done by AGAMAL.

[<Internals\\IDIs health volunteers\\IDI 47 year old Health volunteer-Berwong>](file:///C:\Users\chatio\Desktop\Save%20in%20drive\studies\PK\SMC%20report\Final%20SMC%20report\Volunteers\Diseases%20and%20prevelance\6fdfd973-722e-4f1e-86d3-340409612512) - § 1 reference coded [1.95% Coverage]

Reference 1 - 1.95% Coverage

R: It has reduced.

Q: It has reduced? Is the reduction marginal or a drastic reduction?

R: Ok, that is what I said, I never heard of any child been sick of malaria again, you know am a volunteer if any child has a problem they will inform me I will see to it that whether the child deserves to go to hospital or not if the child is to go to hospital I will go and explain to them what happened and they take care of the child but I haven’t seen that, but since am not a doctor I can conclude that it has gone completely but I will say it has reduced.

[<Internals\\IDIs health volunteers\\IDI-50 year health volunteer-Zambo>](file:///C:\Users\chatio\Desktop\Save%20in%20drive\studies\PK\SMC%20report\Final%20SMC%20report\Volunteers\Diseases%20and%20prevelance\1c65b50f-05fe-4b67-b5d3-3eaa61dbf9c2) - § 1 reference coded [1.60% Coverage]

Reference 1 - 1.60% Coverage

R: there is a big change.

Q: why do you say so?

R: I am saying so because I am a health volunteer in the community and in the past two years mothers were coming to me for malaria drug frequently but if you look at this year I have not seen many mother coming here for malaria drug.
