# Supplementary material for: Community acceptability of Seasonal Malaria Chemoprevention of morbidity and mortality in young children: A qualitative study in the Upper West Region of Ghana
Source: PLoS One. 2019 May 17;14(5):e0216486. doi: 10.1371/journal.pone.0216486 (PMC6524792; doi:10.1371/journal.pone.0216486)
Supplement: S1 File — (ZIP) [file pone.0216486.s001.zip › Study data set-Nvivo coding/Side effects of the drug.docx]

Side effects of the drug

**IDIs with mothers**

[<Internals\\IDIs health workers\\IDIs mothers\\IDI 18 year old mother-Tanziir>](file:///C:\Users\chatio\Desktop\Save%20in%20drive\studies\PK\SMC%20report\Final%20SMC%20report\Mothers\Experiences\d9286b71-0d0b-4b69-a3d3-30b1fce77d91) - § 1 reference coded [0.99% Coverage]

Reference 1 - 0.99% Coverage

Q: What problem did your child encountered when he was given the drug?

R: My child didn’t face any problem at all.

[<Internals\\IDIs health workers\\IDIs mothers\\IDI 20 year old mother-Gbier>](file:///C:\Users\chatio\Desktop\Save%20in%20drive\studies\PK\SMC%20report\Final%20SMC%20report\Mothers\Experiences\6d8a376a-063d-4b88-94d3-30b1fe15cc3a) - § 1 reference coded [0.89% Coverage]

Reference 1 - 0.89% Coverage

Q: Did you record any side effect from your child as a result of taking these malaria drugs?

R: Not at all.

[<Internals\\IDIs health workers\\IDIs mothers\\IDI 20 yearold mother-Berwong1>](file:///C:\Users\chatio\Desktop\Save%20in%20drive\studies\PK\SMC%20report\Final%20SMC%20report\Mothers\Experiences\c8dc56f9-1da6-4c14-88d3-30b1fe3e4370) - § 1 reference coded [0.79% Coverage]

Reference 1 - 0.79% Coverage

Q: When your child took this medicine, has he encountered any problem?

R: No.

[<Internals\\IDIs health workers\\IDIs mothers\\IDI 26 year old mother-Newtown>](file:///C:\Users\chatio\Desktop\Save%20in%20drive\studies\PK\SMC%20report\Final%20SMC%20report\Mothers\Experiences\d2cb5fdf-6b7b-4d12-b6d3-30b1fe4a2a75) - § 1 reference coded [6.15% Coverage]

Reference 1 - 6.15% Coverage

Q. What side reactions have you experienced on your child during the period he was taking the drugs?

R. My child had some rashes that go and come back but I cannot tell whether it was because of the drugs

Q. I want to know whether on the first, second or third days of the drugs that the child experienced the rashes?

R. The child finished the drugs for about a week before I saw the rashes

Q. But were there any side effects on these three days that the child took the drugs?

R. No

[<Internals\\IDIs health workers\\IDIs mothers\\IDI 26 yearold mother-Eremon Tangzu (Autosaved)>](file:///C:\Users\chatio\Desktop\Save%20in%20drive\studies\PK\SMC%20report\Final%20SMC%20report\Mothers\Experiences\dfa639bf-3760-46df-96d3-30b1fe5ad475) - § 1 reference coded [1.31% Coverage]

Reference 1 - 1.31% Coverage

Q: What problem did your child face when he was taking this drug?

R: He did not face any problem taking the drug is his problem.

Q: But did he have fever, vomiting, diarrhea etc the four rounds that he took it?

R: No, he didn’t experience any problem.

[<Internals\\IDIs health workers\\IDIs mothers\\IDI 28 year old mother-Berwong>](file:///C:\Users\chatio\Desktop\Save%20in%20drive\studies\PK\SMC%20report\Final%20SMC%20report\Mothers\Experiences\74cb6986-f6d6-4621-99d3-30b1fe80eacc) - § 2 references coded [1.52% Coverage]

Reference 1 - 0.83% Coverage

R: It was the first day he took the drug that he run diarrhea, he run diarrhea for a week.

Q: He run diarrhea for a week?

R: Yes, but from there he never run diarrhea again.

[<Internals\\IDIs health workers\\IDIs mothers\\IDI 30 year old mother-Eremon Tangzu>](file:///C:\Users\chatio\Desktop\Save%20in%20drive\studies\PK\SMC%20report\Final%20SMC%20report\Mothers\Experiences\99dc5250-fb67-4d4d-8ad3-30b1fea49eb2) - § 1 reference coded [1.00% Coverage]

Reference 1 - 1.00% Coverage

Q: But your child what side effects did he get when he was taken this drug?

R: He didn’t get any side effect.

Q: He didn’t vomit, he didn’t run diarrhea?

R: No.

[<Internals\\IDIs health workers\\IDIs mothers\\IDI 30 year old mother-Gbier>](file:///C:\Users\chatio\Desktop\Save%20in%20drive\studies\PK\SMC%20report\Final%20SMC%20report\Mothers\Experiences\90c36595-6809-4c06-96d3-30b1fee4e4c2) - § 2 references coded [1.71% Coverage]

Reference 1 - 0.93% Coverage

R: When I gave the drugs to the child for the first time. It normally weakened his body.

Reference 2 - 0.78% Coverage

R: The elder one only had body weaken for sometimes and gets active again.

[<Internals\\IDIs health workers\\IDIs mothers\\IDI 30 year old mother-Kolbugnuor>](file:///C:\Users\chatio\Desktop\Save%20in%20drive\studies\PK\SMC%20report\Final%20SMC%20report\Mothers\Experiences\452bf184-0cec-4af6-95d3-30b1feee696f) - § 2 references coded [2.54% Coverage]

Reference 1 - 1.02% Coverage

Q. Did the child experience any side effects from these drugs?

R. No, i have not seen anything

Reference 2 - 1.52% Coverage

R. What i only heard was, some one ever complained of vomiting whenever the child took the drug, but she continued giving the child the drugs

[<Internals\\IDIs health workers\\IDIs mothers\\IDI 30 year old mother-Newtown>](file:///C:\Users\chatio\Desktop\Save%20in%20drive\studies\PK\SMC%20report\Final%20SMC%20report\Mothers\Experiences\1950e505-2542-4323-b9d3-30b1fef7ef36) - § 1 reference coded [1.59% Coverage]

Reference 1 - 1.59% Coverage

Q: Have you encountered any problem/side effects when you gave this medicine to your child?

R: No.

[<Internals\\IDIs health workers\\IDIs mothers\\IDI 30 year old mother-Tuma>](file:///C:\Users\chatio\Desktop\Save%20in%20drive\studies\PK\SMC%20report\Final%20SMC%20report\Mothers\Experiences\713306ab-8ca2-47cb-96d3-30b1feff1321) - § 1 reference coded [6.41% Coverage]

Reference 1 - 6.41% Coverage

R: Ok, the drug, some three tablets were there when I give it to my child he normally vomit everything, this drug they gave as is not like that is was once he vomited, but when he vomited it was one lady and a gentleman who brought it so they gave him another one, apart from that when they give to him he never vomited again. Is this third round that they gave and his temperature was high. I took him to the clinic and they gave him drugs. Those given the SMC drug came the following day to find out how he was doing. They told me to take him to the district hospital if it continue and tell them it is because of the drug he took. But when I gave him the drug he was ok

[<Internals\\IDIs health workers\\IDIs mothers\\IDI 31 year old mother-Eremon Tangzu>](file:///C:\Users\chatio\Desktop\Save%20in%20drive\studies\PK\SMC%20report\Final%20SMC%20report\Mothers\Experiences\07b07256-641a-4fb8-9fd3-30b1ff147f2e) - § 1 reference coded [1.74% Coverage]

Reference 1 - 1.74% Coverage

R**:** No, the first time he took the yellow and the white that he was weak. But when I took him to the clinic, they told me not to be afraid that it is because the malaria is in him and the drug is strong that is why he is weak. They encourage me to still continue to give him that it will go. And when I also continue giving him the drug, it did stop and he became active and ok

[<Internals\\IDIs health workers\\IDIs mothers\\IDI 31 year old mother-Kolbugnuor>](file:///C:\Users\chatio\Desktop\Save%20in%20drive\studies\PK\SMC%20report\Final%20SMC%20report\Mothers\Experiences\e249c295-786b-44e2-96d3-30b1ff33714a) - § 1 reference coded [1.67% Coverage]

Reference 1 - 1.67% Coverage

Q; Can you tell me the problem your child faces when he takes the medicine?

R; He does not face any problem when he takes the medicine.

[<Internals\\IDIs health workers\\IDIs mothers\\IDI 32 year old mother-Gbier>](file:///C:\Users\chatio\Desktop\Save%20in%20drive\studies\PK\SMC%20report\Final%20SMC%20report\Mothers\Experiences\169607a2-22bc-48ff-b4d3-31cc71f08747) - § 2 references coded [1.89% Coverage]

Reference 1 - 1.11% Coverage

Q: what side effects have your child got at the time he was taking this drug?

R: I have not faced any side effects.

Reference 2 - 0.78% Coverage

Q: did you child had fever, vomiting or diarrhea during the medication?

R: no

[<Internals\\IDIs health workers\\IDIs mothers\\IDI 35 year old mother-Bagri>](file:///C:\Users\chatio\Desktop\Save%20in%20drive\studies\PK\SMC%20report\Final%20SMC%20report\Mothers\Experiences\4d2676eb-d04a-4099-a3d3-30b1ff7f9d5c) - § 2 references coded [3.98% Coverage]

Reference 1 - 1.66% Coverage

R: When I started giving the child the medicine I have not encountered any problem.

Q: But like you child if you have given to him and he is sick?

R: For me when I gave to my child he is never sick with any disease.

Reference 2 - 2.32% Coverage

R: The first day he runs diarrhea but it stops in the evening time, so I told them and they said the medicine is cleaning his stomach, apart from that nothing happing again.

Q: What did you do when your child was running diarrhea?

R: I didn’t do anything and it stopped’ I didn’t give him any medicine.

[<Internals\\IDIs health workers\\IDIs mothers\\IDI 35 year old mother-Tanziir>](file:///C:\Users\chatio\Desktop\Save%20in%20drive\studies\PK\SMC%20report\Final%20SMC%20report\Mothers\Experiences\f4a9dcf4-f49d-44b0-9bd3-30b1ff9e8f9d) - § 1 reference coded [1.49% Coverage]

Reference 1 - 1.49% Coverage

R: They are different, the hospital medicine they give when he takes it he normally becomes weak, but this one they brought even if he takes it he does become weak.

[<Internals\\IDIs health workers\\IDIs mothers\\IDI 36 year old mother-Bagri>](file:///C:\Users\chatio\Desktop\Save%20in%20drive\studies\PK\SMC%20report\Final%20SMC%20report\Mothers\Experiences\8687b6b6-f1c5-444b-acd3-30b1ffbfe296) - § 2 references coded [2.18% Coverage]

Reference 1 - 1.19% Coverage

R: For that one no, I only heard that some took the drugs for their children and they run diarrhea, but to me I say they were already sick before taken the drugs.

Reference 2 - 0.99% Coverage

Q: But can you tell me problems encounter when given your child the medicine?

R: No, for me I have not encountered any problem at all.

[<Internals\\IDIs health workers\\IDIs mothers\\IDI 36 year old mother-Ngman-gbil>](file:///C:\Users\chatio\Desktop\Save%20in%20drive\studies\PK\SMC%20report\Final%20SMC%20report\Mothers\Experiences\d09e840c-5e47-4690-a9d3-30b1ffcbc9dc) - § 1 reference coded [0.53% Coverage]

Reference 1 - 0.53% Coverage

R: No, my child has never faced any problem he is healthy.

[<Internals\\IDIs health workers\\IDIs mothers\\IDI 37 year old mother-Kolbugnuor>](file:///C:\Users\chatio\Desktop\Save%20in%20drive\studies\PK\SMC%20report\Final%20SMC%20report\Mothers\Experiences\e04a7c1d-5fa7-4576-8dd3-30b1ffd54e38) - § 3 references coded [3.27% Coverage]

Reference 1 - 0.90% Coverage

R; Some complain their children run diarrhorea.

Reference 2 - 0.75% Coverage

R; Initially he was running diarrhorea.

Reference 3 - 1.62% Coverage

Q; How many days he run diarrhorea?

R; Four days before I sent him to the hospital.

[<Internals\\IDIs health workers\\IDIs mothers\\IDI 50 year old mother-Ngman-gbil>](file:///C:\Users\chatio\Desktop\Save%20in%20drive\studies\PK\SMC%20report\Final%20SMC%20report\Mothers\Experiences\2c4c0db0-dba2-4de3-bcd3-30b1ffdc73e0) - § 2 references coded [2.18% Coverage]

Reference 1 - 1.26% Coverage

R: No it was the younger one they give one day but it was the medicine that was working and the body was weak but in four days time he was ok.

Reference 2 - 0.92% Coverage

Q: It is all the time when they give him the medicine that happened?

R: No it is only the second once.

[<Internals\\IDIs health workers\\IDIs mothers\\IDI mother-Tanziir>](file:///C:\Users\chatio\Desktop\Save%20in%20drive\studies\PK\SMC%20report\Final%20SMC%20report\Mothers\Experiences\9368addf-d833-4882-86d3-30b1ffe5f82d) - § 1 reference coded [1.56% Coverage]

Reference 1 - 1.56% Coverage

R: When my child took the drug nothing bad happened it was only the second one he took and vomited and the body was warm and I took him to the health centre and they took care of him.

**FGDs with fathers and mothers**

[<Internals\\FGDs\\FGD mothers with children under five-Bagri>](a0552c32-4da9-42f3-87d3-2cbc7f4d15ae) - § 2 references coded [1.26% Coverage]

Reference 1 - 0.36% Coverage

No.3

R: For me, my child it was the first day that he was ill but the rest he did not have any problem again.

Reference 2 - 0.90% Coverage

Q: No.3 you said when your child took the drug he got ill. What did you do when he was ill?

No.3

R: I did not do anything.

No.5

R: For me it was the first day when he took it that he was running diarrhea and I took him to the clinic and they gave me ORS, when he took it stopped.

[<Internals\\FGDs\\FGD fathers with children under five-Zambo>](903102be-0a51-4b0e-93d3-3403caff3dbb) - § 5 references coded [6.05% Coverage]

Reference 1 - 1.80% Coverage

R**;** When my child took the medicine on the first day, the following morning he had a running stomach but yet I continued with the medicine and since then he has never tested positive to malaria or fallen sick.

Reference 2 - 1.47% Coverage

R; When my child took the medicine for the first two days, he vomited and I stopped the mother from giving it to him again but since then the child has never fallen sick (FGD father with child under five, Zambo)

Reference 3 - 0.98% Coverage

Q; When the child was vomiting what did you do?

R; I bought medicine for him and that stopped him from vomiting.

Reference 4 - 1.50% Coverage

Q; Apart from this, what else did you do?

R; I went and complained to the volunteer about the reaction of the medicine and she told me she will go and report to the nurses.

Reference 5 - 0.29% Coverage

R; Yes, some their children vomit.

[<Internals\\FGDs\\FGD mothers with children under five-Bagri>](a0552c32-4da9-42f3-87d3-2cbc7f4d15ae) - § 2 references coded [2.67% Coverage]

Reference 1 - 2.31% Coverage

Q: But you can tell me the problems your children get from taking the drug (SP & AQ).

No.9

R: My child was running diarrhea.

Q: For how many days did he run?

R: Three days.

Q: Was it the first month they brought the drug that it happened?

R: Yes the first month.

Q: What about the second month?

R: It didn’t happen again.

Q: What of the third?

R: No.

Q: Was it only diarrhea that worries him?

R: Yes it was only diarrhea.

No.8

R: My child did not get any problem when he took the drug. The only thing that happen was what I told earlier on, the first day that he felt ill apart from that nothing happen to him again.

Q: So how many days did it take him when he was ill?

R: It was the third day they that it stops.

Reference 2 - 0.36% Coverage

No.3

R: For me, my child it was the first day that he was ill but the rest he did not have any problem again.

[<Internals\\FGDs\\FGD mothers with children under five-Zambo>](35ad761a-e06d-40cf-bcd3-3403cb27b45e) - § 5 references coded [2.98% Coverage]

Reference 1 - 0.51% Coverage

R: That diarrhea and vomiting the child was just vomiting and running diarrhea.

Q: Was it when he took the drug?

R: Yes that was the first day he took the drug and they send him to the hospital.

Reference 2 - 0.25% Coverage

R: He normally vomits everything but I believe that because it already entered some remain inside.

Reference 3 - 0.45% Coverage

R: The volunteer said when he vomits we should tell him, so we told him and he gave us another one and we gave him but he vomited again and it continues like that to the end.

Reference 4 - 0.84% Coverage

No.7

R: My child was the first person to take the drug when they brought it, he vomited when they give but it was because they mix the white and yellow for him at once. So when he and asked they said he should give him the white one first and wait for some time and give him the yellow so he did that he never vomited again.

Reference 5 - 0.93% Coverage

No.6

R: For me when I give to my child the first day the following morning my child became weak so I called the volunteer and ask what I should do and she said I should leave him that if it is still like that she knows what to do but for some time if he is ok I should continue to give him, so later he became normal, aside that I never faced any problem again.

[<Internals\\FGDs\\FGD-fathers with children under five-Gbier>](72a70c42-22e7-42d9-a8d3-3ea9fb2b287c) - § 1 reference coded [0.32% Coverage]

Reference 1 - 0.32% Coverage

No.3

R: My child did not experience any problem just as they said.

**IDIs with health volunteers**

[<Internals\\IDIs health volunteers\\IDI 34 year old Health volunteer-Ngman-gbil>](file:///C:\Users\chatio\Desktop\Save%20in%20drive\studies\PK\SMC%20report\Final%20SMC%20report\Volunteers\Opinion%20and%20experience%20on%20intervention\9792d99e-569d-43a4-aad3-3404088aea56) - § 3 references coded [0.66% Coverage]

Reference 1 - 0.14% Coverage

R: Some their bodies itching and some vomit.

Reference 2 - 0.27% Coverage

R: In my community here what I have seen is itching and vomiting and nothing else.

Reference 3 - 0.25% Coverage

R: Yes, they normally say the drug is bitter that they won’t take the drug.

[<Internals\\IDIs health volunteers\\IDI 35 year old Health volunteer-Bagri>](file:///C:\Users\chatio\Desktop\Save%20in%20drive\studies\PK\SMC%20report\Final%20SMC%20report\Volunteers\Opinion%20and%20experience%20on%20intervention\570786a5-e7ba-4707-a3d3-340408a2b74c) - § 2 references coded [1.01% Coverage]

Reference 1 - 0.49% Coverage

R: What we experienced is that children become weak, and some run diarrhea I think these are the only effects.

Reference 2 - 0.52% Coverage

Q: No child vomited?

R: Yes no it is the first round that you always give to every child and the child is vomiting.

[<Internals\\IDIs health volunteers\\IDI 35 year old Health volunteer-Newtown>](file:///C:\Users\chatio\Desktop\Save%20in%20drive\studies\PK\SMC%20report\Final%20SMC%20report\Volunteers\Opinion%20and%20experience%20on%20intervention\e62ddbb9-5b7b-4af3-aed3-340408b8247e) - § 3 references coded [3.39% Coverage]

Reference 1 - 1.35% Coverage

Q. What do people especially mothers with children under five say about these drugs?

R. As i said earlier, some mothers complain because their children vomits or become weak after taken the drug

Reference 2 - 1.41% Coverage

R. We were told to always sleep under treated bed nets

Q. What should someone do when the child experiences the side effects of these drugs?

R. We advise the person to visit the nearest health facility

Reference 3 - 0.64% Coverage

Q. What are the possible side effects of these drugs?

R. Vomiting, skin rashes and weakness

[<Internals\\IDIs health volunteers\\IDI 35 year old Health volunteer-Tuma>](file:///C:\Users\chatio\Desktop\Save%20in%20drive\studies\PK\SMC%20report\Final%20SMC%20report\Volunteers\Opinion%20and%20experience%20on%20intervention\ec8b1f3e-8b41-4285-89d3-340408c8cd1c) - § 3 references coded [1.41% Coverage]

Reference 1 - 0.73% Coverage

R: When someone takes it he/she may be vomiting or itching or become weak, but if someone was having the disease already you will run diarrhea, or be itching or you will have some bulbs all over your body.

Reference 2 - 0.26% Coverage

R: Ok, someone may be ill or shivering, all these show a sign of malaria.

Reference 3 - 0.41% Coverage

R: The problems are that some children took it and were itching, or the eyes swells or had bulbs all over your body.

[<Internals\\IDIs health volunteers\\IDI 36 year old Health volunteer-Tanziir>](file:///C:\Users\chatio\Desktop\Save%20in%20drive\studies\PK\SMC%20report\Final%20SMC%20report\Volunteers\Opinion%20and%20experience%20on%20intervention\1a01ef21-0554-43f3-acd3-340408de3ab4) - § 3 references coded [3.05% Coverage]

Reference 1 - 0.66% Coverage

R: The yellow drugs when you give it the child always want to vomit, so the vomiting is the problem worrying the children. But is not all of them that vomit anyway.

Reference 2 - 1.48% Coverage

R: The side effects the vomiting and diarrhea that I know of, most people said if the child takes the drug that they run diarrhea. So when we go to the training told the health workers that some are saying their children run diarrhea when they take the drug, but they told us that is normally but if the child run for about three days the child have to go to hospital.

Reference 3 - 0.91% Coverage

R: Ok when we go to collect the drug my colleagues normally say many things but I could not hear so that is the only one I know. What I also heard was that a child also got weak but was said to be as a result of an over dose.

[<Internals\\IDIs health volunteers\\IDI 45 year old Health volunteer- Kolbugnuor>](file:///C:\Users\chatio\Desktop\Save%20in%20drive\studies\PK\SMC%20report\Final%20SMC%20report\Volunteers\Opinion%20and%20experience%20on%20intervention\db73d9f0-71e5-4376-86d3-340409449585) - § 2 references coded [0.84% Coverage]

Reference 1 - 0.64% Coverage

R. Some of the problems are, children vomiting after taking the drug and those mothers who are difficult with their children

Reference 2 - 0.21% Coverage

R. Vomiting, dizziness and skin rashes

[<Internals\\IDIs health volunteers\\IDI 45 year old Health volunteer-Gbier>](file:///C:\Users\chatio\Desktop\Save%20in%20drive\studies\PK\SMC%20report\Final%20SMC%20report\Volunteers\Opinion%20and%20experience%20on%20intervention\dfa339b5-1827-441d-9bd3-340409553f66) - § 2 references coded [1.05% Coverage]

Reference 1 - 0.71% Coverage

Q. What side effects do you know of these drugs?

R. They complain of dizziness, vomiting, inching and weakness

Reference 2 - 0.34% Coverage

Q. Is there any side effect?

R. Some do run diarrhea

[<Internals\\IDIs health volunteers\\IDI 47 year old Health volunteer-Berwong>](file:///C:\Users\chatio\Desktop\Save%20in%20drive\studies\PK\SMC%20report\Final%20SMC%20report\Volunteers\Opinion%20and%20experience%20on%20intervention\6fdfd973-722e-4f1e-86d3-340409612512) - § 2 references coded [1.48% Coverage]

Reference 1 - 0.63% Coverage

R: if you give drug to a child to take it means the child should take and be strong and healthy but some are there when they take it worries them example the child can get weak.

Reference 2 - 0.86% Coverage

R: For that if it occurred and they didn’t tell me I can’t know but as a volunteer I told them to inform me if anything of that sort happen, so up there one child suffered fever and they told me and I asked them to take the child to hospital.

[<Internals\\IDIs health volunteers\\IDI-50 year health volunteer-Zambo>](C:\\Users\\chatio\\Desktop\\Save in drive\\studies\\PK\\SMC report\\Final SMC report\\Volunteers\\Opinion and experience on intervention\\1c65b50f-05fe-4b67-b5d3-3eaa61dbf9c2) - § 1 reference coded [0.88% Coverage]

Reference 1 - 0.88% Coverage

R; To me is only one child the mother complain that he sleep through out the night and she send the child to hospital and they admitted him
